# Effects of sleep deprivation on sports performance and perceived exertion in athletes and non-athletes: a systematic review and meta-analysis

**DOI:** 10.3389/fphys.2025.1544286

**Published:** 2025-04-01

**Authors:** Yan Kong, Beibei Yu, Guangming Guan, Yang Wang, Hui He

**Affiliations:** ^1^ Department of Exercise Physiology, Beijing Sport University, Beijing, China; ^2^ DeRucci Healthy Sleep Co., Ltd., Beijing, China; ^3^ China Institute of Sport and Health Science, Beijing Sport University, Beijing, China

**Keywords:** sleep deprivation, aerobic endurance performance, anaerobic sports performance, fatigue, ratings of perceived exertion

## Abstract

**Background:**

Sleep deprivation can significantly affect sports performance and the perception of fatigue. However, the impact of sleep deprivation on sports performance remains a subject of ongoing debate across different populations.

**Objectives:**

This study aimed to investigate the effects of sleep deprivation on sports performance and ratings of perceived exertion (RPE) in different groups, as well as how different types of sleep deprivation affect these aspects.

**Methods:**

This systematic review followed the PRISMA guidelines (PROSPERO CRD42023492792). Randomized controlled trials (RCTs) and randomized crossover studies published in any language or up to any date were eligible based on the P.I.C.O.S. criteria. The systematic search included databases such as PubMed, Cochrane, Embase, Web of Science, and EBSCO, covering studies up to September 2024. The Cochrane RoB 2 tool was used to assess the risk of bias. Meta-analysis was conducted using either a fixed-effect model or a random-effects model. This study conducted subgroup analyses based on different populations, types of sleep deprivation, and testing times.

**Results:**

This review includes 45 studies, comprising 16 on aerobic endurance (AE) performance, 8 on anaerobic endurance (AnE) performance, 23 on explosive power (EP), 10 on maximum force (MF), 4 on speed, 4 on skill control, and 12 on rating of perceived exertion (RPE). The results indicate that sleep deprivation significantly impaired AE in athletes [SMD = −0.66; 95% CI (−1.28, −0.04); *P* = 0.04], as well as EP [SMD = −0.63; 95% CI (−0.94, −0.33); *P* < 0.00001], MF [SMD = −0.35; 95% CI (−0.56, −0.14); *P* = 0.001], speed [SMD = −0.52, 95% CI (−0.83, −0.22); *P* = 0.0008], skill control [SMD = −0.87; 95% CI (−1.7, −0.04); *P* = 0.04], and RPE [SMD = 0.39; 95% CI (0.11, 0.66); *P* = 0.006]. Additionally, AE was also reduced in healthy non-athletes [SMD = −1.02; 95% CI (−1.84, −0.21); *P* = 0.01]. During the sleep deprivation process, early sleep deprivation (PSDE) significantly reduced EP [SMD = −1.04; 95% CI (−1.58, −0.5); *P* = 0.0002], MF [SMD = −0.57; 95% CI (−0.94, −0.19); *P* = 0.003], speed [SMD = −0.78; 95% CI (−1.35, −0.2); *P* = 0.008], and RPE [SMD = 0.6; 95% CI (0.17, 1.02); *P* = 0.006]. Late sleep deprivation (PSDB) impacted speed [SMD = −0.57; 95% CI (−1.15, 0.01); *P* = 0.05], skill control [SMD = −2.12; 95% CI (−3.01, −1.24); *P* < 0.00001], and RPE [SMD = 0.47; 95% CI (0.02, 0.92); *P* = 0.04]. Overall, total sleep deprivation primarily affected AE [SMD = −0.56; 95% CI (−1.08, −0.05); *P* = 0.03]. In terms of testing phases, p.m. tests had a significant impact on AE [SMD = −1.4; 95% CI (−2.47, −0.34); *P* = 0.01], EP [SMD = −0.68; 95% CI (−1.06, −0.31); *P* = 0.0004], MF [SMD = −0.3; 95% CI (−0.51, −0.09); *P* = 0.005], skill control [SMD = −2.12; 95% CI (−3.01, −1.24); *P* < 0.00001], and RPE [SMD = 0.72; 95% CI (0.20, 1.24); *P* = 0.007]. In contrast, a.m. tests primarily affected speed [SMD = −0.81; 95% CI (−1.52, −0.1); *P* = 0.03] and RPE [SMD = 0.44; 95% CI (0.01, 0.86); *P* = 0.04].

**Conclusion:**

Sleep deprivation significantly impairs athletes' performance across various domains, including AE, MF, speed, and skill control, while also exacerbating RPE. In contrast, although sleep deprivation also negatively affects the AE of healthy non-athletes. Furthermore, PSDE appears to have a more pronounced effect on sports performance overall. Additionally, performance assessments conducted in the p.m. have been shown to further impact sports performance. These findings are crucial for understanding how sleep deprivation impacts both athletes and non-athletes, particularly in the context of training and competitive settings.

## 1 Introduction

Sleep is a fundamental human activity during which critical metabolic and cognitive processes, including memory consolidation, occur (Samuels, 2008; Filipas et al., 2021a). However, various real-life factors can reduce sleep duration, leading to both acute and chronic sleep deprivation (Ferrara and De Gennaro, 2001; Lopes et al., 2023). Sleep deprivation is characterized by a consistent reduction or loss of sleep, which can be categorized into total sleep deprivation and partial sleep deprivation. Total sleep deprivation involves the complete elimination of sleep for a specified duration (typically at least one night), resulting in prolonged wakefulness (Reynolds and Banks, 2010). Partial sleep deprivation refers to a reduction in sleep duration below an individual’s typical baseline or the amount required to maintain optimal performance (Reynolds and Banks, 2010). It can be further classified into two types: late sleep deprivation (PSDB) and early sleep deprivation (PSDE) (Gong et al., 2024). Sleep deprivation negatively affects work productivity (Kecklund and Axelsson, 2016), exacerbates fatigue (Goel, 2017), and impairs the performance of daily activities (Vanderlinden et al., 2020). Furthermore, sleep deprivation may serve as a stimulus influencing pre-exercise perceptual difficulties (Reilly, 1983; Magnuson et al., 2023). Consequently, sleep deprivation may elevate ratings of perceived exertion (RPE). Athletes are particularly vulnerable to sleep disturbances due to pre-competition stress, intense training regimens, and the demands of competing across time zones (Juliff et al., 2015; Nedelec et al., 2018). This can lead to impairments in subsequent motor performance (Lastella et al., 2014). Healthy non-athletes may also experience the effects of sleep deprivation due to factors such as life stress. Sleep deprivation impairs athletic performance, including muscle strength, speed (Souissi et al., 2013; Honn et al., 2019; Souissi et al., 2020), as well as overall bodily functions (Craven et al., 2022), and reduces aerobic performance (Craven et al., 2022; Lopes et al., 2023). Sleep deprivation leads to a decline in athletic performance, resulting in impaired neuromuscular coordination, increased injury risk, and delayed recovery in both athletes and non-athletes (Charest and Grandner, 2020). The differential effects of sleep deprivation on sports performance and RPE in athletes versus non-athletes remain unclear, particularly in relation to training level. One study found that PSDE had a more pronounced effect on athletes' selective attention than PSDB (Zerouali et al., 2010). However, it remains unclear how different types of sleep deprivation affect sports performance and RPE in athletes and healthy non-athletes.

Several meta-analyses investigating sleep deprivation as an intervention have compared its effects on exercise performance (Pilcher and Huffcutt, 1996; Gao et al., 2019; Craven et al., 2022; Lopes et al., 2023; Gong et al., 2024). However, the conclusions drawn from these analyses regarding the impact of sleep deprivation on various types of sports performance remain inconclusive. A recent meta-analysis reported that sleep deprivation has a moderate impact on endurance performance (Lopes et al., 2023). In contrast, Craven et al. (2022) found that the extent of sleep deprivation did not influence endurance performance. Acute sleep restriction has been shown to reduce explosive power, speed, and other athletic performance metrics in athletes (Gong et al., 2024). However, a crossover study found that acute sleep restriction had no effect on tennis players' ability to perform repeated sprints (Vitale et al., 2021). While recent meta-analyses have explored the effects of total and partial sleep deprivation on aerobic performance across individuals with different training states (Lopes et al., 2023), as well as the impact of various acute sleep deprivation types on athletic performance (Gong et al., 2024), these studies do not fully capture the effects of sleep deprivation on various aspects of sports performance, nor the differential effects across populations. Furthermore, studies have identified sleep deprivation as a contributing factor to motor perception difficulties (Reilly, 1983; Wang et al., 2021). Motor perception difficulties can also reduce athletes' willingness and efficiency to continue performing (Brotherton et al., 2019a; Roberts et al., 2019b). However, it remains unclear whether this effect extends to non-athletes. Therefore, an updated meta-analysis is needed to examine the effects of sleep deprivation on sports performance and RPE in both athletes and non-athletes, as well as the impact of different types of sleep deprivation on various aspects of sports performance.

The originality of this study lies in its comprehensive investigation of the effects of sleep deprivation on both athletic performance and RPE, with a specific focus on comparing athletes and healthy non-athletes. Unlike previous studies, this systematic review and meta-analysis aims to provide a comprehensive summary of the evidence regarding the impact of sleep deprivation on sports performance and perceived fatigue in both athletes and healthy non-athletes. The secondary aim of this study was to examine the effects of different types of sleep deprivation on sports performance and RPE. We hypothesized that sleep deprivation would have a more pronounced impact on performance and RPE in non-athletes compared to athletes, due to their lower baseline fitness levels and less developed coping mechanisms. Furthermore, we hypothesized that different types of sleep deprivation would have differential effects on specific aspects of performance.

## 2 Methods

### 2.1 Registration

The systematic review and meta-analysis was reported according to Preferred Reporting Items for Systematic Reviews and Meta-analyses (PRISMA) (Shamseer et al., 2015). The study plan was registered in the PROSPERO International Prospective Systematic Review Registry (registration number: CRD42023492792).

### 2.2 Literature search strategy

We searched PubMed, Cochrane, Web of Science and EBSCO databases for experimental studies from inception until publication on 21 September 2024, and the Embase database for relevant studies published in English on 22 September 2024, using the following PICOS criteria: (P) population: human; (I) interventions: sleep deprivation, sleep restriction, or insufficient sleep; (C) control group: normal sleep; (O) outcome measures: aerobic endurance performance, anaerobic endurance performance, explosive power, maximum force, speed, skills control (such as serving accuracy), rating of perceived exertion (RPE), etc.; and (S) study type: experimental studies. To identify relevant studies, we systematically reviewed the reference lists of the selected articles. Additionally, we conducted forward and backward citation searches to ensure a comprehensive coverage of the literature. Forward citation searches involved tracking citations of included studies, while backward citation searches involved examining the reference lists of these studies to identify additional relevant publications. This dual approach was used to minimize the risk of missing pertinent studies (Supplementary Table S1).

### 2.3 Exclusion and inclusion criteria

Studies meeting the following criteria were eligible for inclusion: (1) Research subjects: athletes and non-athletes (athletes, defined as professionals engaged in sports, including elite athletes, amateur athletes and university athletes; non-athletes: healthy subjects except athletes); (2) Experimental research: the study included participants who underwent at least one type of sleep deprivation protocol, such as total sleep deprivation (TSD) (at least one night without sleep) (Banks and Dinges, 2007), partial sleep deprivation at the beginning of the night (PSDB) (falling asleep later than usual, e.g., starting sleep at 3 a.m.), partial sleep deprivation at the end of the night (PSDE) (waking up earlier than the normal waking time, e.g., getting up at 3 a.m.), and partial sleep deprivation of unspecified type (partial). Whether it is a randomized controlled trial or crossover trials. Total sleep deprivation is the complete elimination of sleep gained during a specific period of time (Smithies et al., 2021). Partial sleep deprivation refers to a modest reduction in the amount of sleep you get for one or more nights (getting ∼2–6 h of sleep per night) (Smithies et al., 2021); (3) outcome measures: reporting of at least one of the following exercises related to sporting performance and perception of fatigue: aerobic endurance performance, anaerobic endurance performance, explosive power, maximum force, speed, skills control (such as serving accuracy), RPE, etc.; (4) The sleep deprivation condition must have been compared to a control condition involving a night with habitual sleep time; (5) Only studies published in peer-reviewed journals and written in English were included in our analyses. There was no limit to the studies’ publication date.

We excluded studies that (1) involved unhealthy people, such as those with sleep disorders or other medical conditions that could affect sleep or performance; (2) allowed sleep deprivation to be conducted in conjunction with physical exercise, cognitive tasks, caloric restriction, stimulants or sedatives (e.g., caffeine, L-tryptophan, or modafinil), or taking drugs or placebos were conducted only in one study group; (3) sports performance was measured after a period of recovery sleep (sleep latency tests were not considered ‘recovery sleep’); (4) did not provide outcome measures; (5) were duplicate publications; (6) were literature review papers, letters to the editor, abstracts published in conference proceedings; (7) were animal model studies; (8) sports performance data were not adequately reported (i.e., mean ± standard deviation [SD] was not reported or could not be derived) and contacted by means such as email with no results; or (9) an inter-subject experimental design is allowed, with no baseline measurements after “normal sleep”; (10) Observational study.

### 2.4 Data extraction

All the retrieved studies were imported into the EndNote literature management software. The initial de-duplication was performed using EndNote software, followed by a manual check to ensure accuracy. Two investigators screened the literature based on the inclusion and exclusion criteria. Studies were excluded first by reading the title and abstract and then following a detailed full-text review.

The studies were screened independently by two researchers. Given the objective nature of the screening process and the clear criteria used, no formal inter-rater reliability assessment (e.g., Cohen’s kappa) was conducted. Any disagreements that arose were addressed through open discussion, where the researchers shared their views and reasoning. Consensus was reached after multiple rounds of review and exchange, with the third author stepping in for a final ruling in cases where an agreement could not be reached. The extracted content included (1) basic information about the included studies (such as the name of the first author and publication date), (2) participants’ baseline characteristics (such as age, gender, and number of participants in the experimental and control groups), (3) intervention measures of the experimental group (such as duration and method of sleep intervention), and (4) relevant outcome measures, including aerobic endurance, anaerobic endurance, maximum power, explosive power, speed, skill control, and perceived exertion (RPE), were extracted as part of the outcome data. To ensure the comparability of the data across studies, we adopted a standardized extraction format. For example, the RPE score was recorded using the Borg RPE Scale, following established methods in the literature. Standardized test protocols were also applied to sports performance indicators. Given the diversity of sports performance metrics, any variations in the measurement of the same performance outcomes across different studies were included for comparison. The extracted data were managed using Excel, with all data extraction forms numbered according to the study’s author and publication year, and stored in Excel to ensure data integrity and traceability.

### 2.5 Risk of bias

Two reviewers independently assessed the methodological quality of the included studies. A third reviewer was consulted if disagreements persisted. The methodological quality of RCTs and cross-over studies was evaluated by the Cochrane Collaboration risk of bias (RoB) 2.0 tool (Sterne et al., 2019).

### 2.6 Data synthesis and statistical analysis

For each outcome, the pre-post changes in the experimental and control groups were also pooled to estimate the effects. The outcome measures in this study were continuous variables, and the standard MD (SMD) and its 95% confidence interval (CI) were used as the effect measures. The SMD was used as the effect index. Between-study heterogeneity was measured via *I* ^*2*^ statistics and the Cochran’s Q test (Higgins et al., 2003). The Cochran manual recommends that if *I* ^*2*^ < 50% or Q test *P* > 0.1 and the heterogeneity between different study groups was small, the fixed effect model was used to combine the effect size. If *I* ^*2*^ ≥ 50% or Q test *P* ≤ 0.1, both of which indicate a high degree of heterogeneity, effect sizes were pooled using a random effects model that accounted for between-study variation and weighted each study accordingly. Publication bias was assessed by examining the funnel plot and Egger’s test. If publication bias was indicated, the effect of bias on the obtained results was assessed using the trim-and-fill method (Weinhandl and Duval, 2012). Sensitivity analyses were performed to test the robustness of the pooled results by deleting trials that assessed the risk of bias. The quantitative synthesis of the data was performed using Review Manager5.4 or Stata16.0 software. We analyzed different types of exercise performance and perceived fatigue separately to examine how sleep deprivation affects each aspect of performance and fatigue. To explore how different factors influence the effects of sleep deprivation on athletic performance, subgroup analyses will be conducted based on the following criteria: participant type (athletes vs healthy non-athletes), sleep deprivation type (TSD vs PSDB vs PSDE vs P), and testing time (a.m. vs p.m.).

## 3 Results

### 3.1 Results of the literature search

**FIGURE 1:**
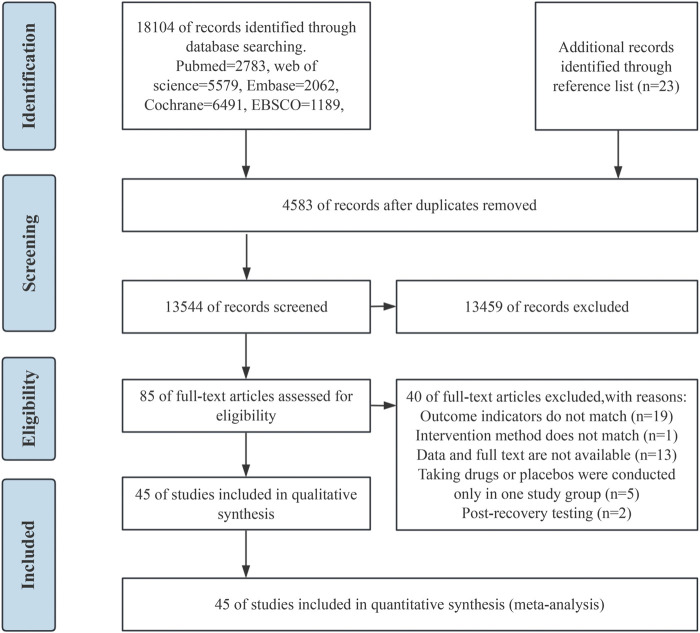
Flowchart of the study selection process.

A preliminary examination identified 18,127 potentially eligible articles, with 18,104 sourced from database searches and 23 from reference lists. After removing 4,583 duplicates, 13,544 articles remained for further screening. Through the process of title and abstract screening, 12,231 articles were excluded. An additional 85 articles were excluded after full-text review, of which 13 lacked full-text or data, and attempts to contact the authors for clarification went unanswered. The remaining 45 articles were included for quantitative synthesis. (Figure 1).

### 3.2 Characteristics of the included studies

Table 1 summarizes the characteristics of the included trials. A total of 670 participants were included; the participants were athletes in 28 studies and healthy non-athletes in 17 studies. Seven studies included female participants, and two study did not specify the ratio of male to female. Data from seven studies were included twice due to differences in sleep intervention duration, population, test methods, and other reasons. The age of the participants was 15–40 years old, and most of them were young people. The sleep deprivation protocols applied in the studies predominantly involved single or dual forms of sleep deprivation (TSD, PSDB, PSDE), with certain studies incorporating a washout period to eliminate the influence of the previous form of deprivation in cases where two forms were used. The control group across all studies adhered to a normal sleep routine.

**TABLE 1:** Basic characteristics of the included studies.

	Population				Sleep deprivation		Test period	Main outcome		Research type
	Author/Year	Subjects	Age	Sex (male/Female)	Type	Duration		Type	Outcome measure	
1	Martin and Chen (1984)	Healthy subjects	25 ± 2	8 (5/3)	TSD	50 h	a.m.	AE	TTE	CT
2	Chen (1991)	Healthy subjects	24.1 ± 2.1	15 (15/0)	TSD	30 h	p.m.	AE	TTE	CT
3	Mougin et al. (1996)	Healthy subjects	19.2 ± 4.95	8 (8/0)	PSDB	6.5 h	a.m.	EP, AnE	WAT	CT
4	Mougin et al. (2001)	athletes	24.0 ± 0.8	8 (8/0)	PSDB	4 h	p.m.	AE	Cycling (10 W.min-1)	CT
5	Mougin et al. (2001)	athletes	24.0 ± 0.8	8 (8/0)	PSDE	4 h	p.m.	AE	Cycling (10 W.min-1)	CT
6	Souissi et al. (2003)	Healthy subjects	22.4 ± 2.4	13 (13/0)	TSD	7.5 h	a.m./p.m.	EP, AnE	WAT	CT
7	Racinais et al. (2004)	athletes	28.1 ± 5.42	22 (17/5)	TSD	38 h	a.m.	AE	Shuttle run score	CT
8	Blumert et al. (2007)	weightlifters	20.7 ± 1.2	9 (9/0)	TSD	1night	a.m.	Fatigue	Borg RPE scale	CT
9	Azboy and Kaygisiz (2009)	middle distant runners	18.1 ± 0.35	10 (10/0)	TSD	1night	a.m.	AE	TTE	CT
10	Azboy and Kaygisiz (2009)	volleyball players	17.8 ± 0.36	10 (10/0)	TSD	1night	a.m.	AE	TTE	CT
11	Ma et al. (2009)	Healthy subjects	20.75 ± 1.18	16 (16/0)	TSD	1night	a.m.	Speed	Reaction Time	CT
12	Oliver et al. (2009)	Healthy subjects	20 ± 3	11 (11/0)	TSD	1night	p.m.	AE	30min endurance running	CT
13	Skein et al. (2011b)	Team sports	21 ± 3	10 (10/0)	TSD	2night	p.m.	AE, MF, EP	30min step-by-step incremental exercise, MVC, sprint	CT
14	Taheri and Arabameri (2012)	Healthy subjects	22.0 ± 1.12	18 (18/0)	TSD	1night	a.m.	EP, AnE, Speed	WAT, RT	CT
15	Abedelmalek et al. (2013)	football players	21.2 ± 1.2	12 (12/0)	PSDE	4 h	a.m./p.m.	EP, AnE	WAT	CT
16	Daanen et al. (2013)	Healthy subjects	21.4 ± 2.5	11 (11/0)	TSD	1night	a.m.	AE, EP, Fatigue	Cycling, VJ, Borg RPE scale	CT
17	HajSalem et al. (2013)	judokas	19.1 ± 1.2	21 (21/0)	PSDE	3 h		EP, AnE, Fatigue	WAT, Borg RPE scale	CT
18	Reyner and Horne (2013)	Tennis	18–22	16 (8/8)	PSDB	2–2.5 h	p.m.	Skill Control	Tennis: 40 serves	CT
19	Konishi et al. (2013)	Healthy subjects	22.6 ± 0.7	10 (10/0)	TSD	1night	p.m.	AE	Running (1 MET.min-1)	CT
20	Skein et al. (2013)	amateur rugby league players	20.4 ± 2.5	11 (11/0)	TSD	1night	a.m.	Fatigue	Borg RPE scale	CT
21	Souissi et al. (2013)	judokas	18.6 ± 2.4	12 (12/0)	PSDB	5 h	a.m./p.m.	MF, EP, AnE, Fatigue	MVC, HG, WAT, Borg RPE scale	CT
22	Souissi et al. (2013)	judokas	18.6 ± 2.5	12 (12/0)	PSDE	5 h	a.m./p.m.	MF, EP, AnE, Fatigue	MVC, HG, WAT, Borg RPE scale	CT
23	Abedelmalek et al. (2014)	football players	21.4 ± 1.4	36	PSDE	4 h	p.m.	EP, AnE	WAT	CT
24	Daviaux et al. (2014)	Healthy subjects	21.4 ± 5.3	24 (12/12)	TSD	24 h	a.m.	MF	MVC	RCT
25	Cincin et al. (2015)	Healthy subjects	33.25 ± 8.78	30 (19/11)	Partial	6 h	a.m.	AE	Running (Bruce)	CT
26	Mejri et al. (2016b)	Taekwondo players	17.6 ± 0.52	10 (10/0)	PSDB	4 h	a.m.	AE, Fatigue	Yo-Yo intermittent test, Borg RPE scale	CT
27	Mejri et al. (2016b)	Taekwondo players	17.6 ± 0.52	10 (10/0)	PSDE	3 h	a.m.	AE, Fatigue	Yo-Yo intermittent test, Borg RPE scale	CT
28	Ben Cheikh et al. (2017)	Karate	16.9 ± 0.8	12	TSD	1 night	a.m.	MF, EP, Speed	MVC and duration, RT	CT
29	Chase et al. (2017)	cyclists	24 ± 7	7 (6/1)	PSDE	4–5 h	a.m.	AE, AnE, Fatigue	3 km timed test (TT), maximal isokinetic torque test, Borg RPE scale	CT
30	Pallesen et al. (2017)	football players	16.5 ± 1.3	19 (19/0)	TSD	1night	a.m.	EP, Skill Control	20 m sprint, 30 m changes direction run juggling, dribbling, controlling	CT
31	Rae et al. (2017)	cyclists	32.3 ± 7.1	16 (16/0)	Partial	3 h	p.m.	EP	Peak power output (PPO) test	CT
32	Brotherton et al. (2019b)	Healthy subjects	22.7 ± 2.5	15 (15/0)	PSDB	4.5 h	p.m.	MF	HG, Bench press, Leg press	CT
33	Cullen et al. (2019)	athletes	27 ± 6	10 (10/0)	TSD	1night	a.m.	AE, MF, EP	Cycling, HG, CMJ	CT
34	Cullen et al. (2019)	athletes	27 ± 6	10 (10/0)	PSDE	3–4 h	a.m.	AE, MF, EP	Cycling, HG, CMJ	CT
35	Daaloul et al. (2019)	Karate	23 ± 2	13 (13/0)	PSDE	4 h	p.m.	EP, Speed	SJ, CMJ, RT	CT
36	Mah et al. (2019)	athletes	28.8 ± 4.51	11 (11/0)	PSDB	3 nights		EP, Speed	DVJ, RT	CT
37	Roberts et al. (2019c)	Bicycle, triathlon	30 ± 6	9 (9/0)	PSDE	3 nights	a.m.	AE, Speed	incremental load test and TT test, RT	CT
38	Roberts et al. (2019b)	Bicycle, triathlon	33 ± 6	13 (13/0)	TSD	1night	a.m.	AE	TT test	CT
39	Romdhani et al., 2019)	judokas	18.5 ± 0.9	14 (14/0)	PSDB	4 h	p.m.	EP, Speed, Fatigue	RSA, RT, Borg RPE scale	CT
40	Romdhani et al. (2019)	judokas	18.5 ± 0.9	14 (14/0)	PSDE	4 h	p.m.	EP, Speed, Fatigue	RSA, RT, Borg RPE scale	CT
41	Ajjimaporn et al. (2020)	football players	20 ± 1	11 (11/0)	PSDE	4 h	p.m.	EP, Fatigue	RSA, Borg RPE scale	CT
42	Romdhani et al. (2020)	judokas	18.78 ± 1.09	9 (9/0)	PSDE	4 h	p.m.	EP, Speed, Fatigue	RSA, RT, Borg RPE scale	CT
43	Skurvydas et al. (2020)	Healthy subjects	20.2 ± 1.4	30 (30/0)	TSD	1 night	a.m.	MF, EP, Speed	HG, CMJ, RT	RCT
44	Souissi et al. (2020)	runner	20.8 ± 1.1	12 (12/0)	Partial	4 h	p.m.	AE, Speed, Fatigue	12 min self-paced running test, Speed, RT Borg RPE scale	CT
45	Filipas et al. (2021a)	basketball players	20 ± 3	19 (19/0)	Partial	5 h	a.m.	Skill Control	Free Throw Basketball Test	CT
46	Skurvydas et al. (2021)	Healthy subjects	20.9 ± 1.32	36 (36/0)	TSD	2night	a.m.	MF	HG, MVC	RCT
47	Vitale et al. (2021)	tennis players	15.4 ± 2.6	12 (12/0)	Partial			AnE, Skill Control	RAS total time, Tennis Technical Performance	CT
48	Knowles et al. (2022)	Healthy subjects	18–35	14 (0/14)	Partial	9 night	a.m.	EP, Fatigue	VJ, Borg RPE sacle	CT
49	Dean et al. (2023)	track-endurance road cyclists	29.9 ± 10.7	10 (10/0)	PSDB	5 h	a.m.	AE, Fatigue	Cycling, Borg RPE scale	CT
50	Saddoud et al. (2023)	Kung-Fu	20.2 ± 1.76	24 (24/0)	TSD	1night	p.m.	EP, MF	seated medicine ball throwing, VJ, IBMS, ILBS	CT
51	Vardar et al. (2007)	Healthy subjects	22.0 ± 1.12	13 (13/0)	TSD	30 h	p.m.	EP, AnE	WAT	CT
52	Vardar et al. (2007)	Healthy subjects	22.0 ± 1.12	13 (13/0)	PSDB	less than 5 h	p.m.	EP, AnE	WAT	CT

Note: AE, aerobic endurance performance; AnE, anaerobic endurance performance; EP, explosive power; MF, maximum force; h, hour; RPE, rating of perceived exertion; VJ, vertical jumps; DVJ, drop maximal vertical jumps; MVC, maximal isometric voluntary contraction; CMJ, counter movement jump; SJ, squat jump; WAT, wingate test; HG, handgrip strength; RSA, repeated sprint ability test; TT, 3 km timed test; TTE, time to exhaustion; RT, reaction time; TSD, total sleep deprivation; PSDB, partial sleep deprivation at the beginning of the night; PSDE, partial sleep deprivation at the end of the night; a.m., ante meridiem, morning; p.m., post meridiem, afternoon; RCT, randomized controlled trials; CT, crossover trials.

To enhance precision in exploring the impact of acute sleep deprivation on diverse athletic abilities and facilitate data integration, this study classified indicators based on their energy supply characteristics and neuromuscular working methods during index tests (Table 2) (Gong et al., 2024).

**TABLE 2:** Types and main characteristics of sports performance and fatigue indicators included in the literature.

Sports performance		Example task
Aerobic endurance (AE)		TTE, 30min endurance running; cycling, etc.
Anaerobic endurance (AnE)		Yo-Yo test total distance, 5msrt total distance, RAS total time, Wingate mean power
strength quality	Maximum Force (MF)	MVC, HG
	Explosive power (EP)	CMJ, SJ, Wingate peak power, VJ, 20 m sprint, RAS peak power (distance)
Speed		5msrt peak distance, Reaction Time (RT)
Skill Control		Juggling, Serve, shootingetc.
rating of perceived exertion		Borg RPE scale

Note: TTE, time to exhaustion; MVC, maximal isometric voluntary contraction; CMJ, counter movement jump; SJ, squat jump; Wingate, Wingate test; HG, handgrip strength; VJ, vertical jumps; 5msrt, 5-m Shuttle Run Test; RSA, repeated sprint ability test; RPE, rating of perceived exertion.

### 3.3 Risk bias assessment results

**FIGURE 2:**
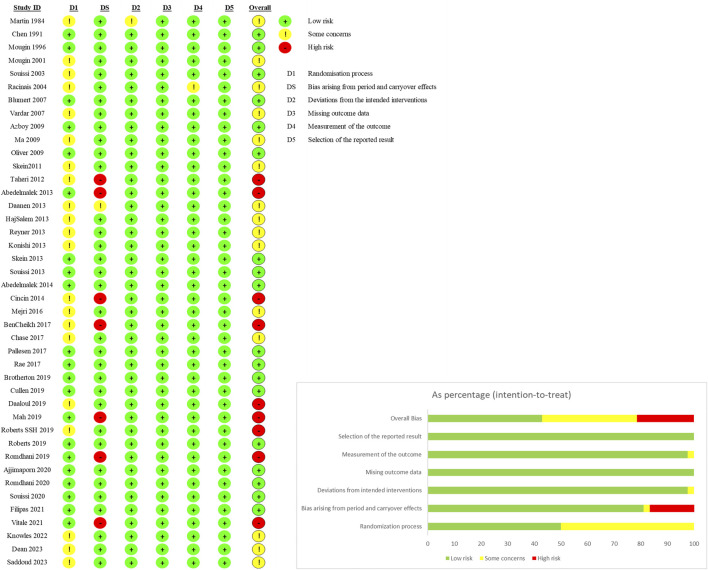
Methodological assessments of cross-over trials by the RoB 2.0 tool.

**FIGURE 3:**
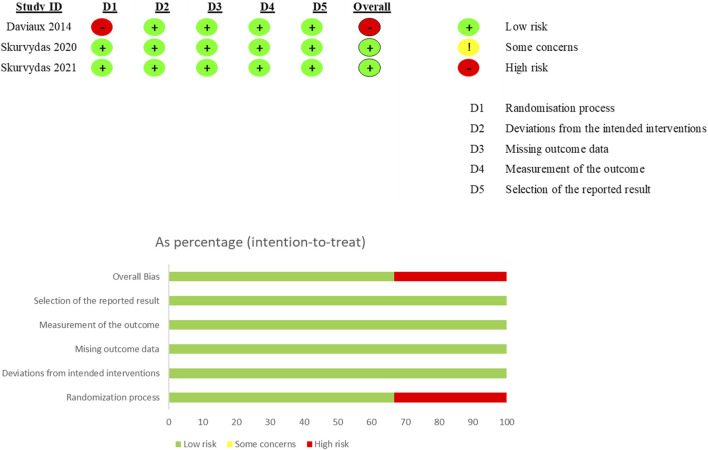
Methodological assessments of randomized controlled trials by the RoB 2.0 tool.

The methodological quality of the included randomized crossover studies and randomized controlled trials (RCTs) is summarized in Figures 2, 3. Among the 42 included crossover studies, 9 have a high risk of bias, 15 have a moderate risk, and 18 have a low risk. Among the three included RCTs, one has a high risk of bias, while two have a low risk of bias. In the crossover studies, the common sources of bias include: (1) unclear randomization process; (2) absence of blinding of assessors; and (3) unclear whether sufficient time was allowed between the first and second phases. The common sources of bias in the RCTs are unclear randomization processes.

### 3.4 Synthesis of the results

#### 3.4.1 Aerobic endurance performance

**FIGURE 4:**
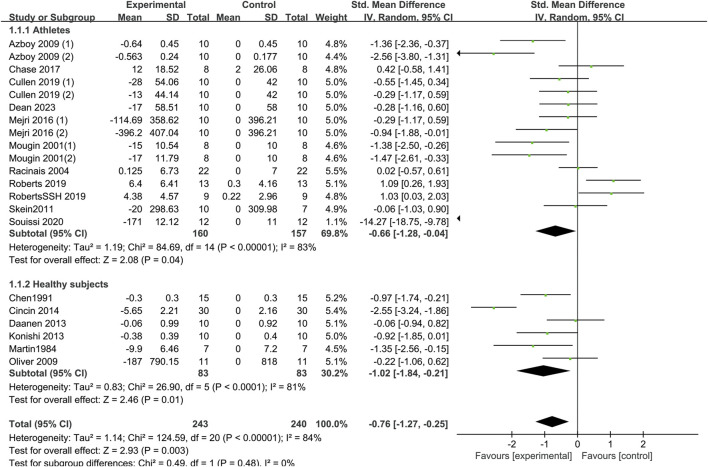
Forest plot of the effect of sleep deprivation on aerobic endurance performance in athletes and healthy non-athletes.

Figure 4 and Supplemetnary Figures S1, S2 Fig present the effects of sleep deprivation on aerobic endurance performance. Using random-effects pooled effect sizes of 16 studies, sleep deprivation significantly reduced aerobic endurance performance compared with control participants [SMD = −0.76; 95% CI (−1.27, −0.25); *P* = 0.003; *I* ^*2*^ = 84%]. Subgroup analysis indicated that sleep deprivation had a statistically significant effect on aerobic endurance performance of athletes [SMD = −0.66; 95% CI (−1.28, −0.04); *P* = 0.04] and healthy non-athletes [SMD = −1.02; 95% CI (−1.84, −0.21); *P* = 0.01]. Moreover, TSD [SMD = −0.56; 95% CI (−1.08, −0.05); *P* = 0.03] caused a decrease in aerobic endurance performance. However, PSDB [SMD = −0.57; 95% CI (−1.22, 0.08); *P* = 0.09], PSDE [SMD = −0.24; 95% CI (−1.08, 0.6); P = 0.58] and Partial [SMD = −8.19; 95% CI (−19.66, 3.28); *P* = 0.16] had no effect on aerobic endurance performance. During the test period, the p.m. tests [SMD = −1.4; 95% CI (−2.47, −0.34); *P* = 0.01] exerted a more pronounced impact on aerobic endurance performance compared to the a.m. tests [SMD = −0.53; 95% CI (−1.12, 0.06); *P* = 0.08], which did not yield any statistically significant effects on aerobic endurance performance.

#### 3.4.2 Anaerobic endurance performance

**FIGURE 5:**
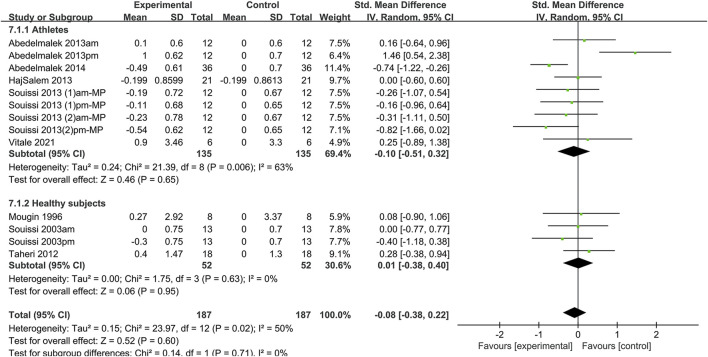
Forest plot of the effect of sleep deprivation on anerobic performance in athletes and healthy non-athletes. am: ante meridiem; pm: post meridiem; MP: Wingate mean power.

The effect of sleep deprivation on anaerobic endurance performance are shown in Figure 5 and Supplemetnary Figures S3, S4. Eight articles assessing the effects of sleep deprivation on anaerobic endurance performance were pooled for analysis. Pooled effect estimates were performed using random effects models. The combined effect size SMD was −0.08 [95% CI (−0.38, 0.22); *P* = 0.6; *I* ^*2*^ = 50%], indicating that the SMD of anaerobic endurance performance was not statistically significant. Subgroup analysis showed that sleep deprivation had no significant effect on the anaerobic endurance performance of athletes [SMD = −0.1, 95% CI (−0.51, 0.32); *P* = 0.65] and healthy non-athletes [SMD = 0.01, 95% CI (−0.38, 0.4); *P* = 0.95]. Upon subgroup analysis of different sleep deprivation types, it was revealed that TSD [SMD = 0, 95% CI (−0.42, 0.42); *P* = 0.99], Partial [SMD = 0.25; 95% CI (−0.89, 1.38); *P* = 0.67], PSDB [SMD = −0.14; 95% CI (−0.63, 0.35); *P* = 0.58], and PSDE [SMD = −0.08; 95% CI (−0.68, 0.51); *P* = 0.79] had no significant impact on anaerobic endurance performance. In subgroup analysis by test time, a.m. tests [SMD = 0.01; 95% CI (−0.31, 0.33); *P* = 0.95] and p.m. tests [SMD = −0.17; 95% CI (−0.89, 0.54); *P* = 0.64] test had no significant effect on anaerobic endurance performance.

#### 3.4.3 Explosive power

**FIGURE 6:**
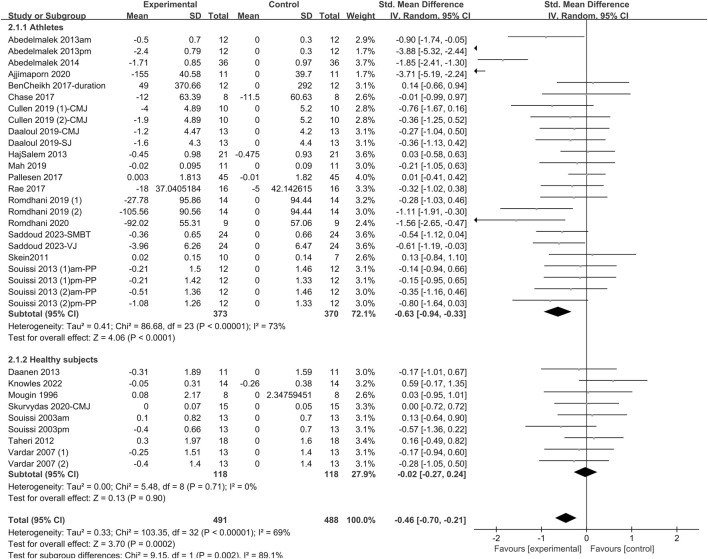
Forest plot of the effect of sleep deprivation on explosive power in athletes and healthy non-athletes. am, ante meridiem; pm, post meridiem; CMJ, Counter Movement Jump; SJ, Squat Jump; VJ, Vertical Jumps; PP, Wingate peak power; SMBT, seated medicine-ball throw.

The effect of sleep deprivation on explosive power are shown in Figure 6 and Supplemetnary Figures S5, S6. A pooled analysis of 23 articles evaluating the effects of sleep deprivation on explosive power was conducted. The random effects model was used to estimate the combined effects. The combined effect size SMD was −0.46 [95% CI (−0.7, −0.21); *P* = 0.0002; *I* ^*2*^ = 69%], indicating that the SMD of explosive power was statistically significant. Subgroup analysis indicated that sleep deprivation intervention significantly altered explosive power in athletes [SMD = −0.63; 95% CI (−0.94, −0.33); *P* < 0.00001], but not of healthy non-athletes [SMD = −0.02; 95% CI (−0.27, 0.24); *P* = 0.9]. Among various types of deprivation, PSDE [SMD = −1.04; 95% CI (−1.58, −0.5); *P* = 0.0002] were found to significantly diminish explosive performance. In contrast, TSD [SMD = −0.18; 95% CI (−0.38, 0.01); *P* = 0.07], PSDB [SMD = −0.19; 95% CI (−0.52, 0.15); *P* = 0.27] and the Partial [SMD = 0.12; 95% CI (−0.77, 1.01); *P* = 0.79] did not exhibit any significant impact on explosive performance. In terms of test period, the p.m. tests [SMD = −0.68; 95% CI (−1.06, −0.31); *P* = 0.0004] markedly altered explosive power, whereas the a.m. tests [SMD = −0.28; 95% CI (−0.6, 0.04); *P* = 0.08] had no significant influence on this aspect of physical performance.

#### 3.4.4 Maximum force

**FIGURE 7:**
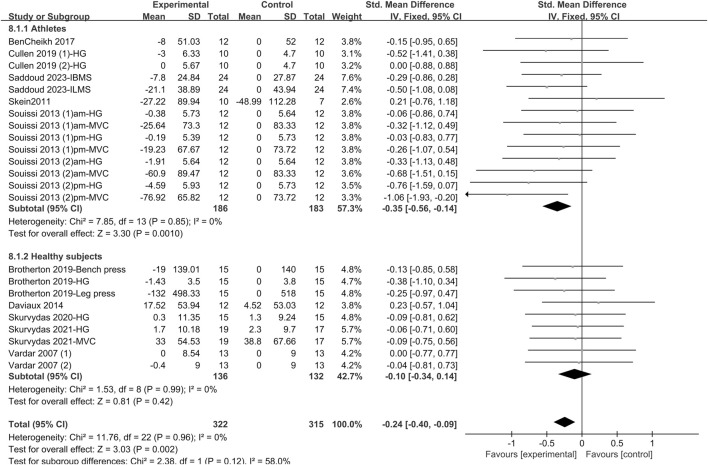
Forest plot of the effect of sleep deprivation on maximum force in athletes and healthy non-athletes. am, ante meridiem; pm, post meridiem; MVC, Maximal Isometric Voluntary Contraction; HG, Handgrip Strength; ILMS, isometric leg-muscles strength; IBMS, isometric back-muscles strength.

The effect of sleep deprivation on maximum force are shown in Figure 7 and Supplemetnary Figures S7, S8. A pooled analysis of 10 articles evaluating the effects of sleep deprivation on maximum force was conducted. The fixed effects model was used to estimate the combined effects. The combined effect size SMD was −0.24 [95% CI (−0.4, −0.09); *P* = 0.002; *I* ^*2*^ = 0%], indicating that the SMD of maximum force was statistically significant. Subgroup analysis indicated that sleep deprivation intervention significantly altered maximum force in athletes [SMD = −0.35; 95% CI (−0.56, −0.14); *P* = 0.001], but not of healthy non-athletes [SMD = −0.1; 95% CI (−0.34, 0.14); *P* = 0.42]. Among various types of deprivation, PSDE [SMD = −0.57; 95% CI (−0.94, −0.19); *P* = 0.003] were found to significantly diminish maximum force performance. In contrast, TSD [SMD = −0.16; 95% CI (−0.39, 0.06); *P* = 0.15] and PSDB [SMD = −0.19; 95% CI (−0.46, 0.08); *P* = 0.17] did not exhibit any significant impact on maximum force performance. In terms of test period, the p.m. tests [SMD = −0.3; 95% CI (−0.51, −0.09); *P* = 0.005] markedly altered maximum force, whereas the a.m. tests [SMD = −0.17; 95% CI (−0.4, 0.06); *P* = 0.15] had no significant influence on this aspect of physical performance.

#### 3.4.5 Speed

**FIGURE 8:**
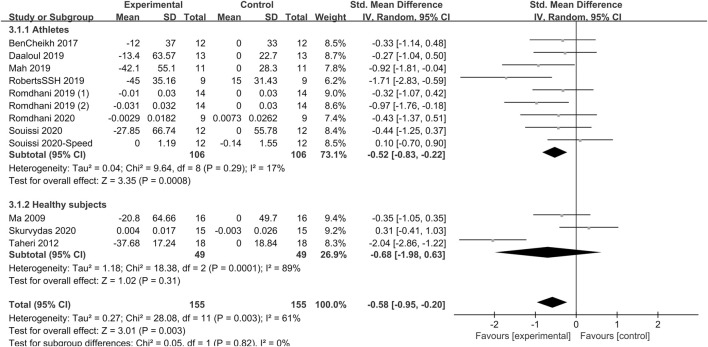
Forest plot of the effect of sleep deprivation on speed performance in athletes and healthy non-athletes.

The effect of sleep deprivation on speed performance are shown in Figure 8 and Supplemetnary Figures S9, S10. Four articles assessing the effects of sleep deprivation on speed performance were pooled for analysis. Pooled effect estimates were performed using random effects models. The combined effect size SMD was −0.58 [95% CI (−0.95, −0.2); *P* = 0.003; *I* ^*2*^ = 61%], indicating that the SMD of speed performance was statistically significant. Subgroup analysis showed that sleep deprivation had a significant effect on the speed performance of athletes [SMD = −0.52, 95% CI (−0.83, −0.22); *P* = 0.0008], but not of healthy non-athletes [SMD = −0.68; 95% CI (−1.98, 0.03); *P* = 0.31]. Upon subgroup analysis of different sleep deprivation types, it was revealed that TSD [SMD = −0.59; 95% CI (−1.54, 0.36); *P* = 0.22] did not exert any significant influence on speed performance, while PSDE [SMD = −0.78; 95% CI (−1.35, −0.2); *P* = 0.008] and PSDB [SMD = −0.57; 95% CI (−1.15, 0.01); *P* = 0.05] significantly reduced speed performance. In the subgroup analysis by test time, the a.m. tests [SMD = −0.81; 95% CI (−1.52, −0.1); *P* = 0.03] markedly altered speed performance, whereas the p.m. tests [SMD = −0.27; 95% CI (−0.63, 0.1); *P* = 0.15] had no significant influence on this aspect of physical performance.

#### 3.4.6 Skill control

**FIGURE 9:**
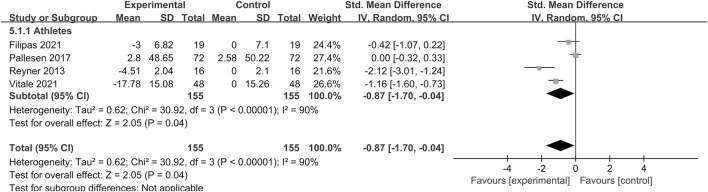
Forest plot of the effect of sleep deprivation on skill control performance in athletes and healthy non-athletes.

The effect of sleep deprivation on skill control are shown in Figure 9 and Supplemetnary Figures S11, S12. Using random-effects pooled effect sizes of four studies, sleep deprivation significantly reduced skill control performance compared with control participants [SMD = −0.87; 95% CI (−1.7, −0.04); *P* = 0.04; *I* ^*2*^ = 90%]. Subgroup analysis showed that sleep deprivation had a significant effect on the skill control of athletes [SMD = −0.87; 95% CI (−1.7, −0.04); *P* = 0.04]. Upon subgroup analysis of different sleep deprivation types, it was revealed that TSD [SMD = 0; 95% CI (−0.32, 0.33); *P* = 0.98] had no significant impact on skill control. Conversely, Partial [SMD = −0.83; 95% CI (−1.55, −0.11); *P* = 0.02], PSDB [SMD = −2.12; 95% CI (−3.01, −1.24); *P* < 0.00001] were found to significantly degrade skill control. In a subgroup analysis based on trial time, a.m. tests [SMD = −0.11; 95% CI (−0.49, 0.26); *P* = 0.55] did not exhibit any significant influence on skill control. In contrast, p.m. tests [SMD = −2.12; 95% CI (−3.01, −1.24); *P* < 0.00001] was found to have an impact on skill control.

#### 3.4.7 Rating of perceived exertion (RPE)

**FIGURE 10:**
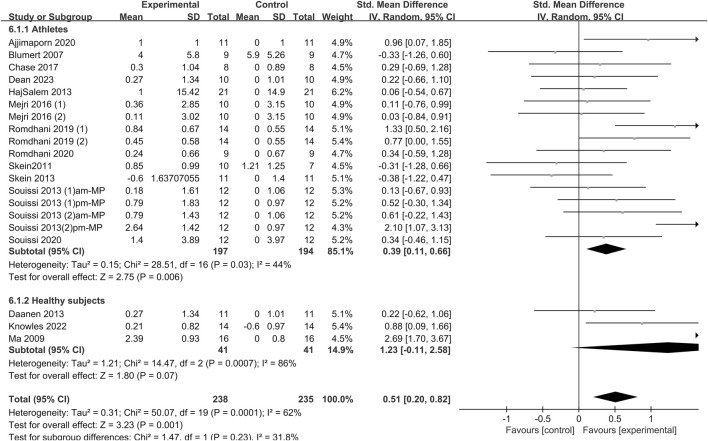
Forest plot of the effect of sleep deprivation on rating of perceived exertion in athletes and healthy non-athletes. am, ante meridiem; pm, post meridiem; MP, Wingate mean power.

The effect of sleep deprivation on RPE are shown in Figure 10 and Supplemetnary Figures S13, S14. By employing random-effects meta-analysis on data from 12 studies, it was determined that sleep deprivation significantly increased the RPE compared to the control group [SMD = 0.51; 95% CI (0.2, 0.82); *P* = 0.001; *I* ^*2*^ = 62%]. Subgroup analyses indicated that while sleep deprivation heightened subjective exertion in athletes [SMD = 0.39; 95% CI (0.11, 0.66); *P* = 0.006], it did not have a similar effect on healthy non-athletes [SMD = 1.23; 95% CI (−0.11, 2.58); *P* = 0.07]. Upon subgroup analysis of different sleep deprivation types, it was revealed that TSD [SMD = 0.37; 95% CI (−0.72, 1.45); *P* = 0.51] had no significant impact on RPE. Conversely, Partial [SMD = 0.62; 95% CI (0.06, 1.18); *P* = 0.03], PSDB [SMD = 0.47; 95% CI (0.02, 0.92); *P* = 0.04], and PSDE [SMD = 0.6; 95% CI (0.17, 1.02); *P* = 0.006] were found to significantly increase RPE. In the time-based subgroup analysis, both a.m. tests [SMD = 0.44; 95% CI (0.01, 0.86); *P* = 0.04] and p.m. tests [SMD = 0.72; 95% CI (0.20, 1.24); *P* = 0.007] increased RPE.

### 3.5 Sensitivity analysis

To confirm the robustness of the results, sensitivity analyses were performed separately for aerobic endurance performance, anaerobic endurance performance, explosive power, maximum force, speed, skills control, RPE. The results revealed that the overall results were robust and stable in sensitivity analyses of aerobic endurance performance, anaerobic endurance performance, explosive power, maximum force, speed, skills control, RPE after deleting each study (Supplementary Figures S15–S21).

### 3.6 Evolution of publication bias

The outcomes of more than 10 RCTs were evaluated for publication bias. No obvious asymmetric distribution was observed in the funnel plot of explosive power, maximum force, speed and RPE, and the Egger’s test showed no publication bias in the correlation analysis between explosive power (t = −2.02, *P* = 0.052), maximum force (t = 0.03, *P* = 0.978), speed (t = −2.03, *P* = 0.07) and RPE (t = 1.24, *P* = 0.229). However, publication bias was noted in the correlation analysis of aerobic endurance performance (t = −2.31, *P* = 0.032). Aerobic endurance performance results were found to be stable after the four virtual articles were supplemented using the trim-and-fill method, indicating that this publishing bias did not affect the estimation (Supplemetnary Figures S22–S27).

## 4 Discussion

In this systematic review and meta-analysis, we synthesize the latest experimental studies on the effects of sleep deprivation on athletic performance and perception of fatigue. Our findings suggest that sleep deprivation significantly reduces the athletes' explosive power, maximum power, speed performance, and motor control, and increases their RPE. For healthy non-athletes, lack of sleep can negatively affect their aerobic endurance performance. However, we observed that sleep deprivation had no significant effect on anaerobic endurance levels in athletes and non-athletes. In addition, our systematic review shows that TSD impairs aerobic endurance performance. PSDE reduces explosive power and maximum force. Both PSDE and PSDB affect speed performance and RPE. PSDB can impair skill control. Regarding the timing of the test, the p.m. assessment had substantial negative effects on aerobic endurance performance, explosive power, maximum force, and skill control. Both a.m. and p.m. tests were associated with an increase in RPE.

### 4.1 Analysis of the effects of sleep deprivation on different types of sports performance

Sleep deprivation exerts a profound and varied impact on sports performance, with different performance indicators being affected to varying extents. The effect size analysis of this study revealed that sleep deprivation had the most significant adverse effects on skill control and aerobic endurance performance, with effect sizes of −0.87 and −0.76, respectively. This was followed by a decline in speed performance and an increase in RPE, with effect sizes of −0.58 and 0.51, respectively. Finally, there was a reduction in explosive power and maximal strength, with effect sizes of −0.46 and −0.24, respectively. Notably, the effect of sleep deprivation on anaerobic endurance was not statistically significant.

The study found that sleep influences various physiological factors, including glucose metabolism (VanHelder et al., 1993), energy metabolism (Penev, 2007), hormone secretion (Leproult and Van Cauter, 2010), and immune function (Santos et al., 2007). The deterioration in skill control motor performance following sleep deprivation is closely linked to impairments in executive functions, including working memory, inhibitory control, and cognitive flexibility (Cheng et al., 2021). Empirical evidence indicates that sleep deprivation leads to prolonged reaction times, increased error rates, and reduced behavioral accuracy (Pallesen et al., 2017; Filipas et al., 2021b). From a neurological perspective, the underlying mechanisms may involve decreased activation of attention and salience networks, which are essential for cognitive resource allocation, and alterations in antagonistic brain networks. This dysfunction is often linked to lower testosterone levels and altered dopamine regulation, as supported by animal models (Filipas et al., 2021b). These findings highlight the complex relationship between sleep deprivation, cognitive function, and neural activity, underscoring the critical role of adequate sleep in preserving optimal cognitive and motor performance (Gong et al., 2024). Aerobic endurance performance is characterized by lower intensity, longer duration (typically more than 30 min), and energy supply predominantly derived from aerobic oxidation (Vondra et al., 1981; Kong et al., 2002). Speed is characterized by high-intensity work, longer duration, and energy primarily derived from glycolysis. Consequently, the availability of energy reserves and the ability to resynthesize energy during exercise are key factors influencing both aerobic endurance and speed performance. Sleep deprivation increases energy expenditure and depletes muscle and liver glycogen, impairing the body’s ability to resynthesize energy substrates during exercise (Skein et al., 2011a). Moreover, sleep is essential for muscle recovery, primarily due to its influence on hormone secretion (Dáttilo et al., 2020). Hormones associated with aerobic endurance performance, such as growth hormone and insulin-like growth factor, are altered following sleep deprivation (Lack and Wright, 2007; Piovezan et al., 2015). As a result, both aerobic endurance and speed performance are impaired following sleep deprivation.

The pronounced impact on explosive power and maximal strength can be attributed to alterations in the RPE, a measure of the subjective effort athletes experience during physical exertion. When athletes perceive the effort required to perform a task as exceeding their maximum capacity, their motivation and efficiency to sustain performance typically decrease (Roberts et al., 2019a). Sleep deprivation can distort the body’s perception of fatigue, leading to heightened subjective fatigue during exercises of comparable intensity (Vitale et al., 2021). This increased fatigue is especially apparent in high-intensity exercises compared to medium-intensity ones (Oliver et al., 2009). Consequently, sleep deprivation affects not only physical capacity but also the mental perception of effort, both of which can significantly influence sports performance and recovery. A study found that mental fatigue had a minor effect on anaerobic performance (Wei et al., 2024), possibly because anaerobic endurance tests require specific power patterns and completion times, resulting in an insignificant total effect size of sleep deprivation on anaerobic endurance performance.

### 4.2 Analysis of the effects of sleep deprivation on sports performance in athletes and healthy non-athletes

The effects of sleep deprivation on athletic performance vary across different populations. The effect size analysis in this study revealed that sleep deprivation significantly impaired athletes' aerobic endurance, explosive power, maximal force, speed, skill control, and RPE, with effect sizes of −0.66, −0.63, −0.35, −0.52, −0.87, and 0.39, respectively. Simultaneously, it negatively affects the aerobic performance of healthy non-athletes, with an effect size of −1.02.

The effects of sleep deprivation on athletes are closely linked to the specific demands of their sport disciplines. In sports that require aggressive and defensive maneuvers against opponents, athletes must possess high levels of physical fitness, including explosiveness, speed, well-developed cognitive functions, sharp decision-making skills, and quick reflexes (Mori et al., 2002; Chaabène et al., 2012; Di Corrado et al., 2014; Daaloul et al., 2019). In contrast, sports that require high precision and skill place a greater emphasis on advanced cognitive performance (Filipas et al., 2021a). Healthy non-athletes, who do not engage in such physically demanding activities regularly, rely less on these physiological parameters in their daily routines. Therefore, explosive power, maximal strength, speed, skill control, and RPE may be more significantly negatively affected in athletes after sleep deprivation than in non-athletes. Regarding aerobic exercise, sleep deprivation increases energy expenditure, making aerobic performance in both athletes and non-athletes susceptible to its detrimental effects.

### 4.3 Analysis of the influence of TSD, PSDE, PSDB on different sports performance

Different aspects of sports performance are influenced by distinct types of sleep deprivation. TSD exerts a more significant effect on aerobic performance (−0.56), while partial sleep deprivation disproportionately affects explosive power, maximal strength, speed, skill control, and RPE. Specifically, explosive power, maximal force, and speed were primarily affected by PSDE (effect sizes −1.04, −0.57, and −0.78, respectively). Skill control was primarily affected by PSDB (effect sizes −0.57 and −2.12, respectively), while RPE was influenced by both PSDB and PSDE (effect sizes 0.47 and 0.6, respectively). Speed performance was also impacted by PSDB and PSDE, with effect sizes of −0.57 and −0.78, respectively. These findings underscore the subtle effects of sleep deprivation on various components of sports performance.

Aerobic endurance performance and explosive power are particularly compromised during total sleep deprivation, likely due to increased energy expenditure and impaired resynthesis of energy substrates. During PSDE, explosive power and maximal force are diminished. Moreover, PSDE has a more pronounced effect on speed performance than PSDB. Zerouali’s research suggests that PSDE has a more pronounced impact on athletes' selective attention than PSDB, possibly due to its effect on rapid eye movement (REM) sleep and slow wave sleep (SWS), critical stages for attention restoration and various physiological functions, such as memory consolidation, immune repair, energy recovery, and hormone release (Zerouali et al., 2010; Mejri et al., 2016a). PSDB may lead to reduced skill control, potentially stemming from difficulty focusing and sustaining attention during PSDB(Boksem et al., 2005). Additionally, reduced testosterone levels following PSDB could affect dopaminergic signaling in brain regions associated with cognition, potentially resulting in an increased incidence of technical errors (Leproult and Van Cauter, 2011; Jardí et al., 2018; Moreira et al., 2018). The effect size of PSDE on RPE is larger than that of PSDB. This discrepancy may be attributed to the prolonged wakefulness and subsequent sleepiness following PSDE (Souissi et al., 2013). Furthermore, research indicates that PSDE is associated with increased inflammation, leading to greater cellular damage and, consequently, heightened subjective sleepiness (Irwin et al., 2016).

### 4.4 Analysis of differences in a.m. and p.m. sports performance after sleep deprivation

The effect size analysis of this study revealed a decrease in speed performance and a significant increase in RPE during the a.m. tests, with effect sizes of −0.81 and 0.44, respectively. In the p.m. tests, aerobic endurance performance, explosive power, maximal force, skill control, and RPE decreased, with effect sizes of −1.4, −0.68, −0.3, −2.12, and 0.72, respectively.

The analysis suggested that sleep deprivation induced fatigue in the subjects, leading to a decrease in alertness, alterations in energy expenditure, depletion of strength reserves, and a decline in motor control precision (Gong et al., 2024). These factors likely contributed to the observed decline in afternoon sports performance following sleep deprivation. Speed performance may be associated with an increase in RPE (Helms et al., 2016). Longer waking hours reduce alertness, prolong reaction time, and decrease speed. However, for RPE, its effect size increased in both morning and afternoon sessions, possibly due to disruption of the circadian rhythm and alteration of muscle cell function following sleep deprivation, which affects muscle contraction and relaxation. Additionally, the extended waking hours compared to a normal night’s sleep lead to a progressive increase in sleep pressure, disrupting sleep homeostasis (Gong et al., 2024). Adenosine accumulation in the brain serves as the physiological foundation for regulating sleep homeostasis (Romdhani et al., 2021). The longer one remains awake, the higher the adenosine concentration, which in turn heightens the perception of exertion following physical activity (Gong et al., 2024).

In conclusion, sleep deprivation affects the athletic performance of both athletes and healthy non-athletes. Although this study primarily focused on the effects of sleep deprivation on sports performance and perceived fatigue, sleep recovery strategies play a key role in mitigating its negative impact. Sleep recovery plays an essential role in restoring cognitive function, muscle recovery, and overall performance (Nédélec et al., 2015; Mesas et al., 2023). Current sleep interventions aimed at recovery include sleep extension, daytime naps, sleep hygiene, and post-exercise recovery strategies (Bonnar et al., 2018; Mesas et al., 2023). These strategies have varying effects on athletic performance and recovery. Therefore, to protect the sleep health and athletic performance of both athletes and non-athletes, appropriate sleep recovery strategies should be adopted following sleep deprivation. It is important to note that while this study primarily focuses on performance testing under standardized laboratory conditions, there may be differences between these tests and an athlete’s performance in real-world sports or competition settings. Laboratory tests provide a controlled environment for assessing the fundamental physiological and psychological factors affecting performance, while real-world performance is influenced by additional uncontrollable factors, such as competitive pressure and environmental conditions. Thus, while the results of this study provide valuable insights into the effects of sleep deprivation on athlete performance, the external validity of these findings in actual sporting contexts may be limited. Future research could further assess the generalizability of these results by conducting sleep deprivation tests in simulated competitive environments or real-world sports settings. For example, evaluating athlete fatigue and recovery based on task performance in actual competitions would help enhance the ecological validity of the research, providing a more accurate representation of performance in real-world athletic situations.

### 4.5 Limitations

This study has several limitations. First, some studies did not provide original data, which prevented the inclusion of all available research in the analysis. Second, due to the limited number of studies, the results for some outcome measures exhibited heterogeneity, making interpretation of the findings challenging. Third, due to the inclusion criteria, the majority of the studies primarily focused on the acute effects of sleep deprivation on sports performance and perceived fatigue, meaning this review is more suited for understanding the short-term physiological impacts of sleep deprivation on sports performance and lacks insights into long-term effects. The studies included in this review utilized a variety of methodologies, including differences in experimental designs, outcome measures, and assessment tools. Fourth, this heterogeneity may introduce some inconsistencies in the interpretation of the results. Despite efforts to standardize data collection and reporting, the variation in methodologies could limit the comparability of studies and the overall robustness of our conclusions. Fifth, one of the key limitations of this meta-analysis is the variability in the control of sleep quality across the included studies. While we selected studies that implemented sleep control nights with habitual sleep patterns and a sleep duration of approximately 7 h per night, there was considerable variation in how sleep quality parameters such as sleep efficiency, sleep latency, and sleep fragmentation were monitored and reported. Some studies did not provide detailed information on these factors, which could potentially influence the results related to sleep deprivation’s effects on athletic performance and fatigue perception. Finally, because there were fewer female participants in the included studies, gender differences could not be analyzed.

## 5 Conclusions and suggestions

Our findings indicate that sleep deprivation significantly impairs aerobic endurance, muscular strength, speed, and motor control, while also increasing perceived exertion (RPE). Specifically, athletes experience significant declines in performance, particularly in aerobic endurance, muscular strength, speed, motor control, and perceived exertion. Similarly, non-athletes also show a notable reduction in aerobic endurance. Further analysis reveals that the impact of sleep deprivation on sports performance extends beyond physical decline and is associated with disruptions in neurological, metabolic, and psychological functions. Notably, the effects of sleep deprivation on performance were more pronounced during assessments conducted in the afternoon and for the PSDE sleep deprivation type. Overall, sleep deprivation can have a broad negative impact on both athletes' and non-athletes' performance, particularly during training or competitive events. Therefore, ensuring adequate sleep is crucial for optimizing athletes' performance and overall health. Future research should explore the specific effects of sleep deprivation on sports performance across different types of exercise and athlete states.

## Data Availability

The original contributions presented in the study are included in the article/Supplementary Material, further inquiries can be directed to the corresponding author.
