# Supplementary material for: Effects of sleep deprivation on sports performance and perceived exertion in athletes and non-athletes: a systematic review and meta-analysis
Source: Front Physiol. 2025 Apr 1;16:1544286. doi: 10.3389/fphys.2025.1544286 (PMC11996801; doi:10.3389/fphys.2025.1544286)
Supplement: Supplementary file 1 [file DataSheet1.pdf]

Supporting information

S1 Table Search strategy for PubMed, Cochrane, Embase, Web of Science and EBSCO database.

**Database**	**Search strategy**
PubMed (2783)	((human[Title/Abstract]) OR (healthy individuals[Title/Abstract]) OR (athlete[Title/Abstract]) OR (player[Title/Abstract]) OR (elite althletes[Title/Abstract]) OR (competitive athlete[Title/Abstract]) OR (sportswoman[Title/Abstract]) OR (sportsman[Title/Abstract])) AND ((sleep[Title/Abstract]) OR (sleep deprivation[Title/Abstract]) OR (sleep restriction[Title/Abstract]) OR (sleep loss[Title/Abstract]) OR (sleep quality[Title/Abstract]) OR (sleep duration[Title/Abstract]) OR (Insufficient sleep[Title/Abstract])) AND ((performance[Title/Abstract]) OR (competition[Title/Abstract]) OR ("technical skills"[Title/Abstract]) OR (tactical[Title/Abstract]) OR (strength[Title/Abstract]) OR (anaerobic[Title/Abstract]) OR (aerobic[Title/Abstract]) OR (accuracy[Title/Abstract]) OR (coordination[Title/Abstract]) OR (flexibility[Title/Abstract]) OR (balance[Title/Abstract]) OR (speed[Title/Abstract]) OR (endurance[Title/Abstract]) OR (RPE[Title/Abstract]) OR (ratings of perceived exertion [Title/Abstract]))
Cochrane (6491)	#1 (human):ti,ab,kw or (healthy individuals):ti,ab,kw or (athlete):ti,ab,kw or (player):ti,ab,kw or (elite althletes):ti,ab,kw or (competitive athlete):ti,ab,kw or (sportswoman):ti,ab,kw or (sportsman):ti,ab,kw<br>#2 (sleep):ti,ab,kw or (sleep deprivation):ti,ab,kw or (sleep restriction):ti,ab,kw or (sleep loss):ti,ab,kw or (sleep quality):ti,ab,kw or (sleep duration):ti,ab,kw or (Insufficient sleep):ti,ab,kw<br>#3 (performance):ti,ab,kw or (competition):ti,ab,kw or (technical skills):ti,ab,kw or (tactical):ti,ab,kw or (strength):ti,ab,kw or (anaerobic):ti,ab,kw or (aerobic):ti,ab,kw or (accuracy):ti,ab,kw or (coordination):ti,ab,kw or (flexibility):ti,ab,kw or (balance):ti,ab,kw or (speed):ti,ab,kw or (endurance):ti,ab,kw or (RPE):ti,ab,kw or (ratings of perceived exertion):ti,ab,kw<br>#4 #1 and #2 and #3
Embase (2062)	#1 'human':ab,ti OR 'healthy individuals':ab,ti OR 'athlete':ab,ti OR 'player':ab,ti OR 'elite althletes':ab,ti OR 'competitive athlete':ab,ti OR 'sportswoman':ab,ti OR 'sportsman':ab,ti

	#2 'sleep':ab,ti OR 'sleep deprivation':ab,ti OR 'sleep restriction':ab,ti OR 'sleep loss':ab,ti OR 'sleep quality':ab,ti OR 'sleep duration':ab,ti OR 'Insufficient sleep':ab,ti<br>#3 'performance':ab,ti OR 'competition':ab,ti OR 'technical skills':ab,ti OR 'tactical':ab,ti OR 'strength':ab,ti OR 'anaerobic':ab,ti OR 'aerobic':ab,ti OR 'accuracy':ab,ti OR 'coordination':ab,ti OR 'flexibility':ab,ti OR 'balance':ab,ti OR 'speed':ab,ti OR 'endurance':ab,ti OR 'RPE':ab,ti OR ' ratings of perceived exertion ':ab,ti<br>#4 #1 AND #2 AND #3
Web of Science (5579)	1: TS= (human) OR (healthy individuals) OR (athlete) OR (player) OR (elite althletes) OR (competitive athlete) OR (sportswoman) OR (sportsman)<br>2: TS= (sleep) OR (sleep deprivation) OR (sleep restriction) OR (sleep loss) OR (sleep quality) OR (sleep duration) OR (Insufficient sleep)<br>3: TS= (performance) OR (competition) OR ("technical skills") OR (tactical) OR (strength) OR (anaerobic) OR (aerobic) OR (accuracy) OR (coordination) OR (flexibility) OR (balance) OR (speed) OR (endurance) OR (RPE) OR (ratings of perceived exertion)<br>5: #1 AND #2 AND #3
EBSCO (1189)	AB (human OR healthy individuals OR athlete OR player OR elite althletes OR competitive athlete OR sportswoman OR sportsman) AND AB (sleep OR sleep deprivation OR sleep restriction OR sleep loss OR sleep quality OR sleep duration OR Insufficient sleep) AND AB (performance OR competition OR "technical skills" OR tactical OR strength OR anaerobic OR aerobic OR accuracy OR coordination OR flexibility OR balance OR speed OR endurance OR RPE OR ratings of perceived exertion)

	Experimental			Control				Std. Mean Difference
Study or Subgroup	Mean	SD	Total	Mean	SD	Total	Weight	IV, Random, 95% CI
**1.2.1 Total**								
Azboy 2009 (1)	-0.64	0.45	10	0	0.45	10	4.8%	-1.36 [-2.36, -0.37]
Azboy 2009 (2)	-0.563	0.24	10	0	0.177	10	4.4%	-2.56 [-3.80, -1.31]
Chen1991	-0.3	0.3	15	0	0.3	15	5.2%	-0.97 [-1.74, -0.21]
Cullen 2019 (1)	-28	54.06	10	0	42	10	5.0%	-0.55 [-1.45, 0.34]
Daanen 2013	-0.06	0.99	10	0	0.92	10	5.1%	-0.06 [-0.94, 0.82]
Konishi 2013	-0.38	0.39	10	0	0.4	10	5.0%	-0.92 [-1.85, 0.01]
Martin1984	-9.9	6.46	7	0	7.2	7	4.5%	-1.35 [-2.56, -0.15]
Oliver 2009	-187	790.15	11	0	818	11	5.1%	-0.22 [-1.06, 0.62]
Racinais 2004	0.125	6.73	22	0	7	22	5.5%	0.02 [-0.57, 0.61]
Roberts 2019	6.4	6.41	13	0.3	4.16	13	5.1%	1.09 [0.26, 1.93]
Skein2011	-20	298.63	10	0	309.98	7	4.9%	-0.06 [-1.03, 0.90]
**Subtotal (95% CI)**			**128**			**125**	**54.6%**	**-0.56 [-1.08, -0.05]**
Heterogeneity: Tau² = 0.53; Chi² = 36.44, df = 10 (P < 0.0001); I² = 73%								
Test for overall effect: Z = 2.16 (P = 0.03)								
**1.2.2 Partial**								
Cincin 2014	-5.65	2.21	30	0	2.16	30	5.4%	-2.55 [-3.24, -1.86]
Souissi 2020	-171	12.12	12	0	11	12	1.1%	-14.27 [-18.75, -9.78]
**Subtotal (95% CI)**			**42**			**42**	**6.4%**	**-8.19 [-19.66, 3.28]**
Heterogeneity: Tau² = 65.92; Chi² = 25.58, df = 1 (P < 0.00001); I² = 96%								
Test for overall effect: Z = 1.40 (P = 0.16)								
**1.2.3 PSDB**								
Dean 2023	-17	58.51	10	0	58	10	5.0%	-0.28 [-1.16, 0.60]
Mejri 2016 (1)	-114.69	358.62	10	0	396.21	10	5.0%	-0.29 [-1.17, 0.59]
Mougin 2001(1)	-15	10.54	8	0	10	8	4.6%	-1.38 [-2.50, -0.26]
**Subtotal (95% CI)**			**28**			**28**	**14.7%**	**-0.57 [-1.22, 0.08]**
Heterogeneity: Tau² = 0.09; Chi² = 2.79, df = 2 (P = 0.25); I² = 28%								
Test for overall effect: Z = 1.72 (P = 0.09)								
**1.2.4 PSDE**								
Chase 2017	12	18.52	8	2	26.06	8	4.8%	0.42 [-0.58, 1.41]
Cullen 2019 (2)	-13	44.14	10	0	42	10	5.0%	-0.29 [-1.17, 0.59]
Mejri 2016 (2)	-396.2	407.04	10	0	396.21	10	5.0%	-0.94 [-1.88, -0.01]
Mougin 2001(2)	-17	11.79	8	0	10	8	4.6%	-1.47 [-2.61, -0.33]
RobertsSSH 2019	4.38	4.57	9	0.22	2.96	9	4.8%	1.03 [0.03, 2.03]
**Subtotal (95% CI)**			**45**			**45**	**24.3%**	**-0.24 [-1.08, 0.60]**
Heterogeneity: Tau² = 0.66; Chi² = 14.52, df = 4 (P = 0.006); I² = 72%								
Test for overall effect: Z = 0.55 (P = 0.58)								
**Total (95% CI)**			**243**			**240**	**100.0%**	**-0.76 [-1.27, -0.25]**
Heterogeneity: Tau² = 1.14; Chi² = 124.59, df = 20 (P < 0.00001); I² = 84%								
Test for overall effect: Z = 2.93 (P = 0.003)								
Test for subgroup differences: Chi² = 2.20, df = 3 (P = 0.53), I² = 0%								

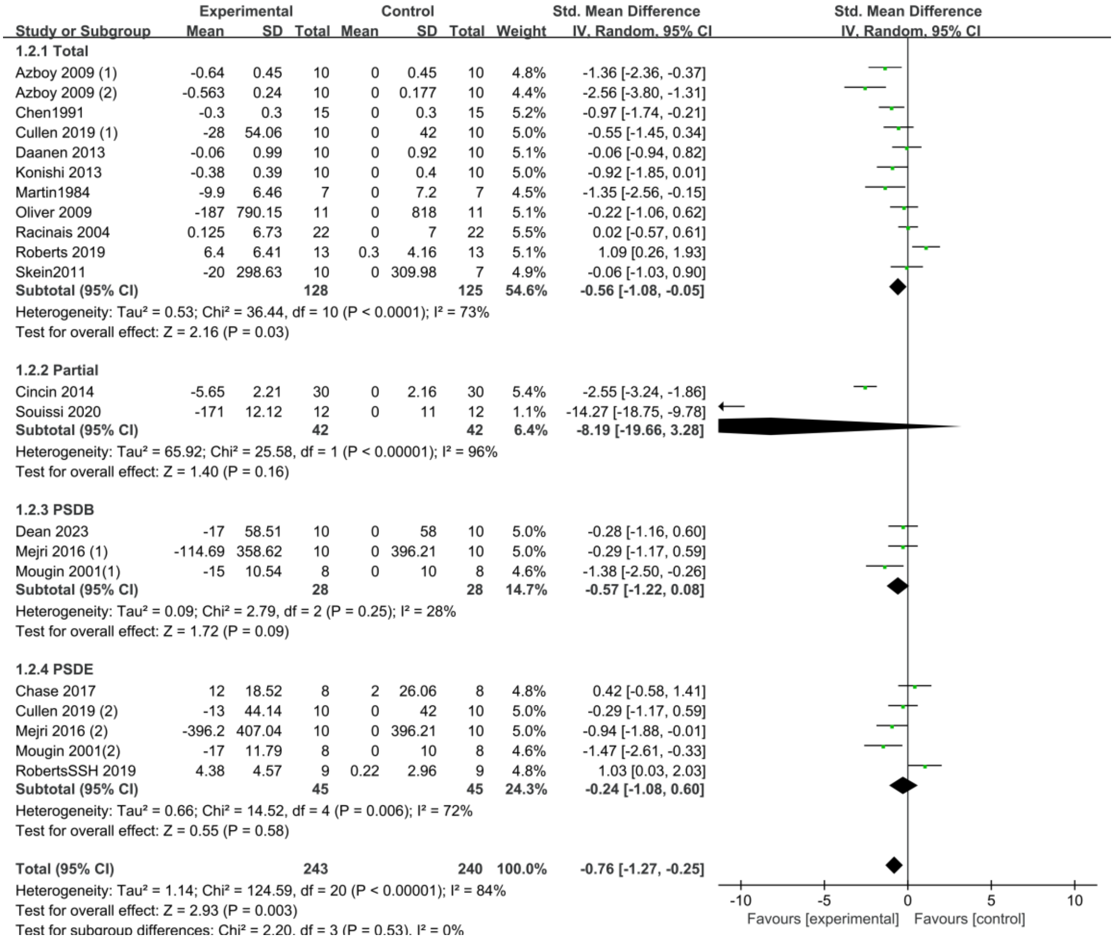

S1 Fig Forest plot of the effects of different types of sleep deprivation on aerobic endurance performance

	Experimental			Control				Std. Mean Difference
Study or Subgroup	Mean	SD	Total	Mean	SD	Total	Weight	IV, Random, 95% CI
**1.3.1 a.m.**								
Azboy 2009 (1)	-0.64	0.45	10	0	0.45	10	4.8%	-1.36 [-2.36, -0.37]
Azboy 2009 (2)	-0.563	0.24	10	0	0.177	10	4.4%	-2.56 [-3.80, -1.31]
Chase 2017	12	18.52	8	2	26.06	8	4.8%	0.42 [-0.58, 1.41]
Cincin 2014	-5.65	2.21	30	0	2.16	30	5.4%	-2.55 [-3.24, -1.86]
Cullen 2019 (1)	-28	54.06	10	0	42	10	5.0%	-0.55 [-1.45, 0.34]
Cullen 2019 (2)	-13	44.14	10	0	42	10	5.0%	-0.29 [-1.17, 0.59]
Daanen 2013	-0.06	0.99	10	0	0.92	10	5.1%	-0.06 [-0.94, 0.82]
Dean 2023	-17	58.51	10	0	58	10	5.0%	-0.28 [-1.16, 0.60]
Martin1984	-9.9	6.46	7	0	7.2	7	4.5%	-1.35 [-2.56, -0.15]
Mejri 2016 (1)	-114.69	358.62	10	0	396.21	10	5.0%	-0.29 [-1.17, 0.59]
Mejri 2016 (2)	-396.2	407.04	10	0	396.21	10	5.0%	-0.94 [-1.88, -0.01]
Racinais 2004	0.125	6.73	22	0	7	22	5.5%	0.02 [-0.57, 0.61]
Roberts 2019	6.4	6.41	13	0.3	4.16	13	5.1%	1.09 [0.26, 1.93]
RobertsSSH 2019	4.38	4.57	9	0.22	2.96	9	4.8%	1.03 [0.03, 2.03]
**Subtotal (95% CI)**			**169**			**169**	**69.5%**	**-0.53 [-1.12, 0.06]**
Heterogeneity: Tau² = 1.05; Chi² = 80.84, df = 13 (P < 0.00001); I² = 84%								
Test for overall effect: Z = 1.75 (P = 0.08)								
**1.3.2 p.m.**								
Chen1991	-0.3	0.3	15	0	0.3	15	5.2%	-0.97 [-1.74, -0.21]
Konishi 2013	-0.38	0.39	10	0	0.4	10	5.0%	-0.92 [-1.85, 0.01]
Mougin 2001(1)	-15	10.54	8	0	10	8	4.6%	-1.38 [-2.50, -0.26]
Mougin 2001(2)	-17	11.79	8	0	10	8	4.6%	-1.47 [-2.61, -0.33]
Oliver 2009	-187	790.15	11	0	818	11	5.1%	-0.22 [-1.06, 0.62]
Skein2011	-20	298.63	10	0	309.98	7	4.9%	-0.06 [-1.03, 0.90]
Souissi 2020	-171	12.12	12	0	11	12	1.1%	-14.27 [-18.75, -9.78]
**Subtotal (95% CI)**			**74**			**71**	**30.5%**	**-1.40 [-2.47, -0.34]**
Heterogeneity: Tau² = 1.60; Chi² = 41.15, df = 6 (P < 0.00001); I² = 85%								
Test for overall effect: Z = 2.59 (P = 0.010)								
**Total (95% CI)**			**243**			**240**	**100.0%**	**-0.76 [-1.27, -0.25]**
Heterogeneity: Tau² = 1.14; Chi² = 124.59, df = 20 (P < 0.00001); I² = 84%								
Test for overall effect: Z = 2.93 (P = 0.003)								
Test for subgroup differences: Chi² = 1.99, df = 1 (P = 0.16), I² = 49.6%								

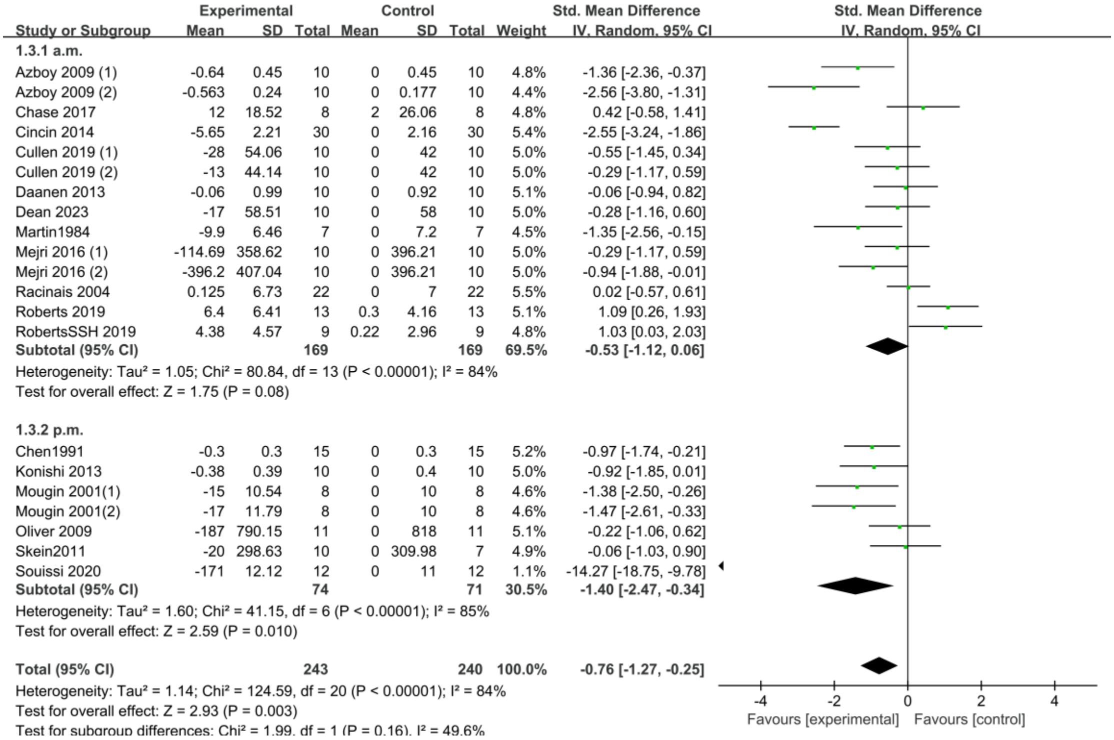

S2 Fig Forest plot of the effects of different test period of sleep deprivation on aerobic endurance performance

Study or Subgroup	Experimental Mean	Experimental SD	Experimental Total	Control Mean	Control SD	Control Total	Weight	Std. Mean Difference IV, Random, 95% CI
**7.2.1 Total**								
Souissi 2003am	0	0.75	13	0	0.7	13	7.8%	0.00 [-0.77, 0.77]
Souissi 2003pm	-0.3	0.75	13	0	0.7	13	7.7%	-0.40 [-1.18, 0.38]
Taheri 2012	0.4	1.47	18	0	1.3	18	9.1%	0.28 [-0.38, 0.94]
**Subtotal (95% CI)**			**44**			**44**	**24.6%**	**-0.00 [-0.42, 0.42]**
Heterogeneity: Tau² = 0.00; Chi² = 1.73, df = 2 (P = 0.42); I² = 0%								
Test for overall effect: Z = 0.01 (P = 0.99)								
**7.2.2 Partial**								
Vitale 2021	0.9	3.46	6	0	3.3	6	4.9%	0.25 [-0.89, 1.38]
**Subtotal (95% CI)**			**6**			**6**	**4.9%**	**0.25 [-0.89, 1.38]**
Heterogeneity: Not applicable								
Test for overall effect: Z = 0.42 (P = 0.67)								
**7.2.3 PSDB**								
Mougin 1996	0.27	2.92	8	0	3.37	8	5.9%	0.08 [-0.90, 1.06]
Souissi 2013 (1)am-MP	-0.19	0.72	12	0	0.67	12	7.5%	-0.26 [-1.07, 0.54]
Souissi 2013 (1)pm-MP	-0.11	0.68	12	0	0.65	12	7.5%	-0.16 [-0.96, 0.64]
**Subtotal (95% CI)**			**32**			**32**	**20.9%**	**-0.14 [-0.63, 0.35]**
Heterogeneity: Tau² = 0.00; Chi² = 0.29, df = 2 (P = 0.87); I² = 0%								
Test for overall effect: Z = 0.55 (P = 0.58)								
**7.2.4 PSDE**								
Abedelmalek 2013am	0.1	0.6	12	0	0.6	12	7.5%	0.16 [-0.64, 0.96]
Abedelmalek 2013pm	1	0.62	12	0	0.7	12	6.4%	1.46 [0.54, 2.38]
Abedelmalek 2014	-0.49	0.61	36	0	0.7	36	11.4%	-0.74 [-1.22, -0.26]
HajSalem 2013	-0.199	0.8599	21	-0.199	0.8613	21	9.7%	0.00 [-0.60, 0.60]
Souissi 2013 (2)am-MP	-0.23	0.78	12	0	0.67	12	7.5%	-0.31 [-1.11, 0.50]
Souissi 2013(2)pm-MP	-0.54	0.62	12	0	0.65	12	7.1%	-0.82 [-1.66, 0.02]
**Subtotal (95% CI)**			**105**			**105**	**49.6%**	**-0.08 [-0.68, 0.51]**
Heterogeneity: Tau² = 0.41; Chi² = 20.74, df = 5 (P = 0.0009); I² = 76%								
Test for overall effect: Z = 0.27 (P = 0.79)								
**Total (95% CI)**			**187**			**187**	**100.0%**	**-0.08 [-0.38, 0.22]**
Heterogeneity: Tau² = 0.15; Chi² = 23.97, df = 12 (P = 0.02); I² = 50%								
Test for overall effect: Z = 0.52 (P = 0.60)								
Test for subgroup differences: Chi² = 0.44, df = 3 (P = 0.93), I² = 0%								

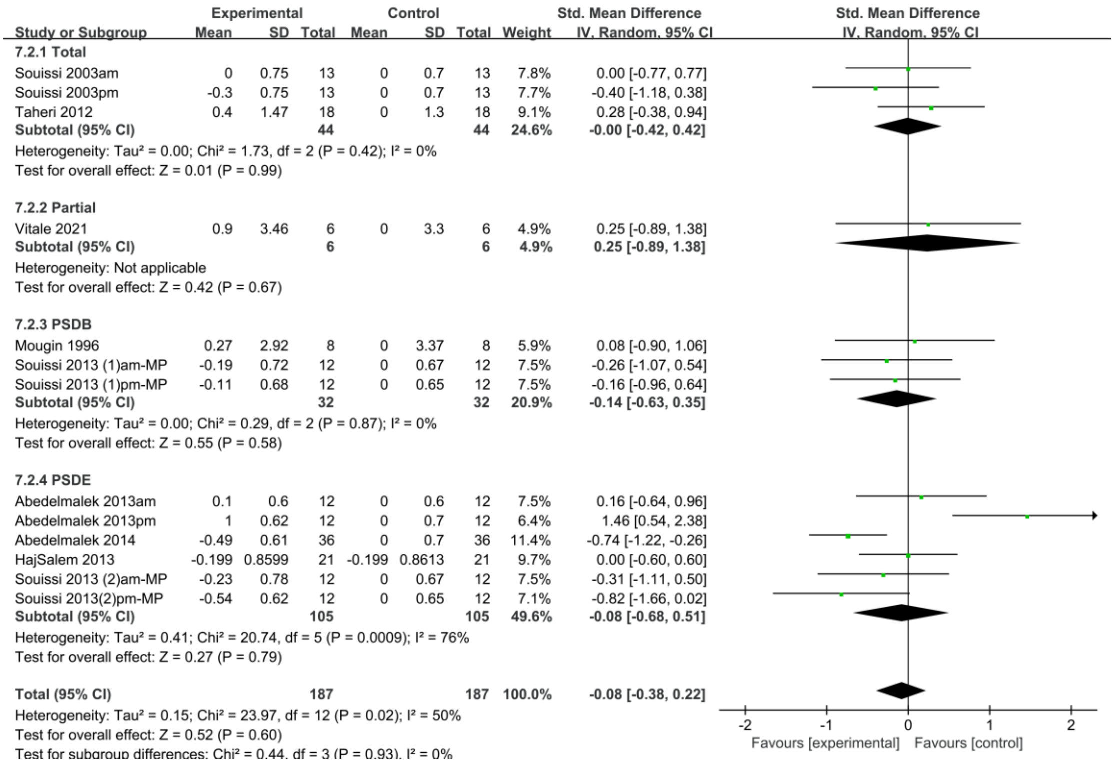

S3 Fig Forest plot of the effects of different types of sleep deprivation on anaerobic endurance performance. am: ante meridiem; pm: post meridiem; MP: Wingate mean power

Study or Subgroup	Experimental Mean	Experimental SD	Experimental Total	Control Mean	Control SD	Control Total	Weight	Std. Mean Difference IV, Random, 95% CI
**7.3.1 a.m.**								
Abedelmalek 2013am	0.1	0.6	12	0	0.6	12	7.5%	0.16 [-0.64, 0.96]
Mougin 1996	0.27	2.92	8	0	3.37	8	5.9%	0.08 [-0.90, 1.06]
Souissi 2003am	0	0.75	13	0	0.7	13	7.8%	0.00 [-0.77, 0.77]
Souissi 2013 (1)am-MP	-0.19	0.72	12	0	0.67	12	7.5%	-0.26 [-1.07, 0.54]
Souissi 2013 (2)am-MP	-0.23	0.78	12	0	0.67	12	7.5%	-0.31 [-1.11, 0.50]
Taheri 2012	0.4	1.47	18	0	1.3	18	9.1%	0.28 [-0.38, 0.94]
**Subtotal (95% CI)**			**75**			**75**	**45.2%**	**0.01 [-0.31, 0.33]**
Heterogeneity: Tau² = 0.00; Chi² = 1.85, df = 5 (P = 0.87); I² = 0%								
Test for overall effect: Z = 0.07 (P = 0.95)								
**7.3.2 p.m.**								
Abedelmalek 2013pm	1	0.62	12	0	0.7	12	6.4%	1.46 [0.54, 2.38]
Abedelmalek 2014	-0.49	0.61	36	0	0.7	36	11.4%	-0.74 [-1.22, -0.26]
Souissi 2003pm	-0.3	0.75	13	0	0.7	13	7.7%	-0.40 [-1.18, 0.38]
Souissi 2013 (1)pm-MP	-0.11	0.68	12	0	0.65	12	7.5%	-0.16 [-0.96, 0.64]
Souissi 2013(2)pm-MP	-0.54	0.62	12	0	0.65	12	7.1%	-0.82 [-1.66, 0.02]
**Subtotal (95% CI)**			**85**			**85**	**40.2%**	**-0.17 [-0.89, 0.54]**
Heterogeneity: Tau² = 0.52; Chi² = 18.88, df = 4 (P = 0.0008); I² = 79%								
Test for overall effect: Z = 0.47 (P = 0.64)								
**7.3.3 without reference to**								
HajSalem 2013	-0.199	0.8599	21	-0.199	0.8613	21	9.7%	0.00 [-0.60, 0.60]
Vitale 2021	0.9	3.46	6	0	3.3	6	4.9%	0.25 [-0.89, 1.38]
**Subtotal (95% CI)**			**27**			**27**	**14.6%**	**0.05 [-0.48, 0.59]**
Heterogeneity: Tau² = 0.00; Chi² = 0.14, df = 1 (P = 0.71); I² = 0%								
Test for overall effect: Z = 0.20 (P = 0.84)								
**Total (95% CI)**			**187**			**187**	**100.0%**	**-0.08 [-0.38, 0.22]**
Heterogeneity: Tau² = 0.15; Chi² = 23.97, df = 12 (P = 0.02); I² = 50%								
Test for overall effect: Z = 0.52 (P = 0.60)								
Test for subgroup differences: Chi² = 0.27, df = 2 (P = 0.87), I² = 0%								

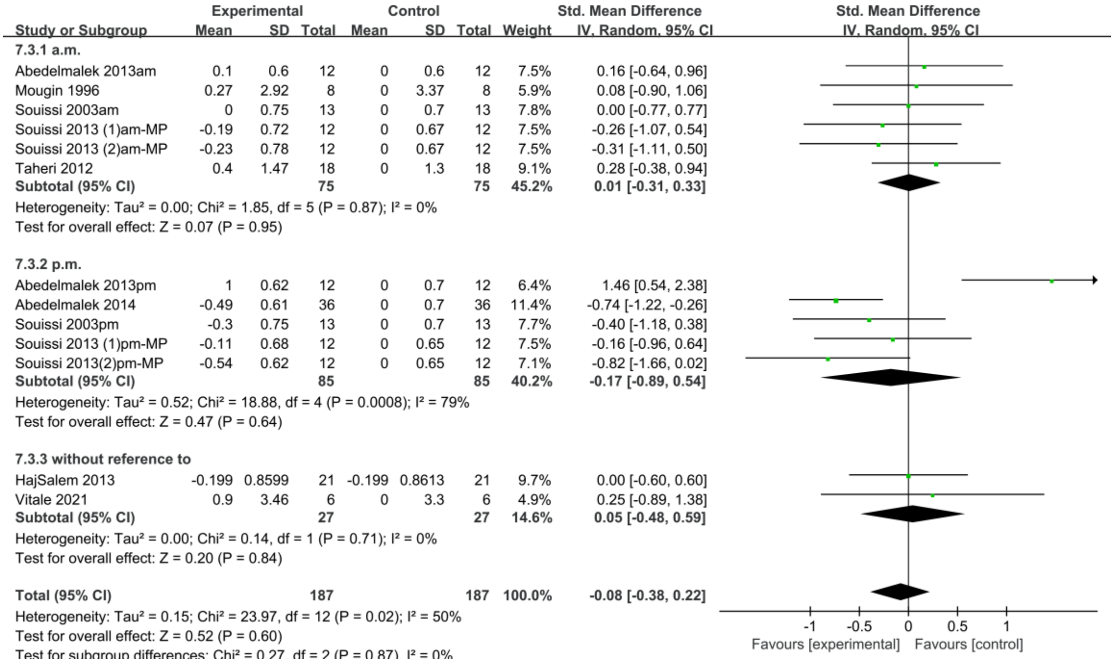

S4 Fig Forest plot of the effects of different test period of sleep deprivation on anerobic endurance performance. am: ante meridiem; pm: post meridiem; MP: Wingate mean power

Study or Subgroup	Experimental Mean	Experimental SD	Experimental Total	Control Mean	Control SD	Control Total	Weight	Std. Mean Difference IV, Random, 95% CI
**2.2.1 Total**								
BenCheikh 2017-duration	49	370.66	12	0	292	12	3.1%	0.14 [-0.66, 0.94]
Cullen 2019 (1)-CMJ	-4	4.89	10	0	5.2	10	2.8%	-0.76 [-1.67, 0.16]
Daanen 2013	-0.31	1.89	11	0	1.59	11	3.0%	-0.17 [-1.01, 0.67]
Pallesen 2017	0.003	1.813	45	-0.01	1.82	45	4.0%	0.01 [-0.41, 0.42]
Saddoud 2023-SMBT	-0.36	0.65	24	0	0.66	24	3.6%	-0.54 [-1.12, 0.04]
Saddoud 2023-VJ	-3.96	6.26	24	0	6.47	24	3.6%	-0.61 [-1.19, -0.03]
Skein2011	0.02	0.15	10	0	0.14	7	2.6%	0.13 [-0.84, 1.10]
Skurvydas 2020-CMJ	0	0.07	15	0	0.05	15	3.3%	0.00 [-0.72, 0.72]
Souissi 2003am	0.1	0.82	13	0	0.7	13	3.1%	0.13 [-0.64, 0.90]
Souissi 2003pm	-0.4	0.66	13	0	0.7	13	3.1%	-0.57 [-1.36, 0.22]
Taheri 2012	0.3	1.97	18	0	1.6	18	3.4%	0.16 [-0.49, 0.82]
Vardar 2007 (1)	-0.25	1.51	13	0	1.4	13	3.1%	-0.17 [-0.94, 0.60]
**Subtotal (95% CI)**			**208**			**205**	**38.8%**	**-0.18 [-0.38, 0.01]**
Heterogeneity: Tau² = 0.00; Chi² = 9.82, df = 11 (P = 0.55); I² = 0%								
Test for overall effect: Z = 1.83 (P = 0.07)								
**2.2.2 Partial**								
Knowles 2022	-0.05	0.31	14	-0.26	0.38	14	3.2%	0.59 [-0.17, 1.35]
Rae 2017	-18	37.0405184	16	-5	42.142615	16	3.3%	-0.32 [-1.02, 0.38]
**Subtotal (95% CI)**			**30**			**30**	**6.5%**	**0.12 [-0.77, 1.01]**
Heterogeneity: Tau² = 0.27; Chi² = 2.97, df = 1 (P = 0.08); I² = 66%								
Test for overall effect: Z = 0.27 (P = 0.79)								
**2.2.3 PSDB**								
Mah 2019	-0.02	0.095	11	0	0.09	11	3.0%	-0.21 [-1.05, 0.63]
Mougin 1996	0.08	2.17	8	0	2.34759451	8	2.6%	0.03 [-0.95, 1.01]
Romdhani 2019 (1)	-27.78	95.86	14	0	94.44	14	3.2%	-0.28 [-1.03, 0.46]
Souissi 2013 (1)am-PP	-0.21	1.5	12	0	1.46	12	3.1%	-0.14 [-0.94, 0.66]
Souissi 2013 (1)pm-PP	-0.21	1.42	12	0	1.33	12	3.1%	-0.15 [-0.95, 0.65]
Vardar 2007 (2)	-0.4	1.4	13	0	1.4	13	3.1%	-0.28 [-1.05, 0.50]
**Subtotal (95% CI)**			**70**			**70**	**18.0%**	**-0.19 [-0.52, 0.15]**
Heterogeneity: Tau² = 0.00; Chi² = 0.34, df = 5 (P = 1.00); I² = 0%								
Test for overall effect: Z = 1.09 (P = 0.27)								
**2.2.4 PSDE**								
Abedelmalek 2013am	-0.5	0.7	12	0	0.3	12	2.9%	-0.90 [-1.74, -0.05]
Abedelmalek 2013pm	-2.4	0.79	12	0	0.3	12	1.7%	-3.88 [-5.32, -2.44]
Abedelmalek 2014	-1.71	0.85	36	0	0.97	36	3.7%	-1.85 [-2.41, -1.30]
Ajjimaporn 2020	-155	40.58	11	0	39.7	11	1.7%	-3.71 [-5.19, -2.24]
Chase 2017	-12	63.39	8	-11.5	60.63	8	2.6%	-0.01 [-0.99, 0.97]
Cullen 2019 (2)-CMJ	-1.9	4.89	10	0	5.2	10	2.8%	-0.36 [-1.25, 0.52]
Daaloul 2019-CMJ	-1.2	4.47	13	0	4.2	13	3.1%	-0.27 [-1.04, 0.50]
Daaloul 2019-SJ	-1.6	4.3	13	0	4.4	13	3.1%	-0.36 [-1.13, 0.42]
HajSalem 2013	-0.45	0.98	21	-0.475	0.93	21	3.6%	0.03 [-0.58, 0.63]
Romdhani 2019 (2)	-105.56	90.56	14	0	94.44	14	3.0%	-1.11 [-1.91, -0.30]
Romdhani 2020	-92.02	55.31	9	0	57.06	9	2.4%	-1.56 [-2.65, -0.47]
Souissi 2013 (2)am-PP	-0.51	1.36	12	0	1.46	12	3.0%	-0.35 [-1.16, 0.46]
Souissi 2013 (2)pm-PP	-1.08	1.26	12	0	1.33	12	3.0%	-0.80 [-1.64, 0.03]
**Subtotal (95% CI)**			**183**			**183**	**36.7%**	**-1.04 [-1.58, -0.50]**
Heterogeneity: Tau² = 0.77; Chi² = 63.24, df = 12 (P < 0.00001); I² = 81%								
Test for overall effect: Z = 3.79 (P = 0.0002)								
**Total (95% CI)**			**491**			**488**	**100.0%**	**-0.46 [-0.70, -0.21]**
Heterogeneity: Tau² = 0.33; Chi² = 103.35, df = 32 (P < 0.00001); I² = 69%								
Test for overall effect: Z = 3.70 (P = 0.0002)								
Test for subgroup differences: Chi² = 9.58, df = 3 (P = 0.02), I² = 68.7%								

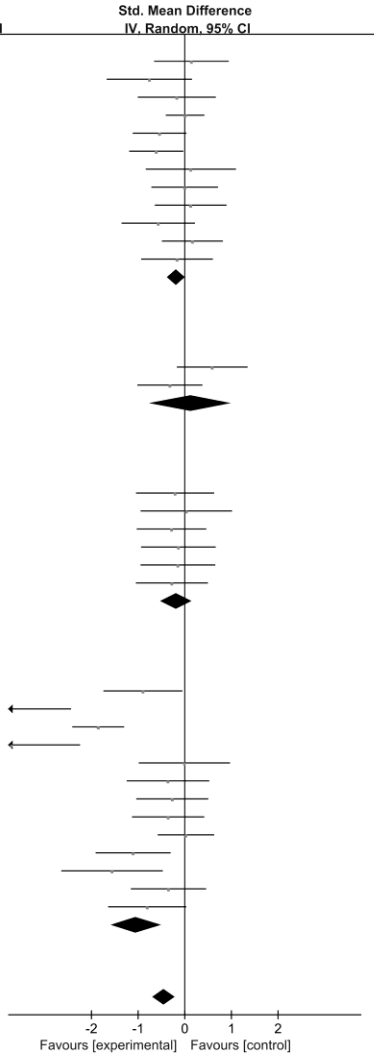

S5 Fig Forest plot of the effects of different types of sleep deprivation on explosive power. am: ante meridiem; pm: post meridiem; CMJ: Counter Movement Jump; SJ, Squat Jump; VJ, Vertical Jumps; PP: Wingate peak power; SMBT: seated medicine-ball throw

Study or Subgroup	Experimental Mean	Experimental SD	Experimental Total	Control Mean	Control SD	Control Total	Weight	Std. Mean Difference IV, Random, 95% CI
**2.3.1 a.m.**								
Abedelmalek 2013am	-0.5	0.7	12	0	0.3	12	2.9%	-0.90 [-1.74, -0.05]
Ajjimaporn 2020	-155	40.58	11	0	39.7	11	1.7%	-3.71 [-5.19, -2.24]
BenCheikh 2017-duration	49	370.66	12	0	292	12	3.1%	0.14 [-0.66, 0.94]
Chase 2017	-12	63.39	8	-11.5	60.63	8	2.6%	-0.01 [-0.99, 0.97]
Cullen 2019 (1)-CMJ	-4	4.89	10	0	5.2	10	2.8%	-0.76 [-1.67, 0.16]
Cullen 2019 (2)-CMJ	-1.9	4.89	10	0	5.2	10	2.8%	-0.36 [-1.25, 0.52]
Daanen 2013	-0.31	1.89	11	0	1.59	11	3.0%	-0.17 [-1.01, 0.67]
Knowles 2022	-0.05	0.31	14	-0.26	0.38	14	3.2%	0.59 [-0.17, 1.35]
Mougin 1996	0.08	2.17	8	0	2.34759451	8	2.6%	0.03 [-0.95, 1.01]
Pallesen 2017	0.003	1.813	45	-0.01	1.82	45	4.0%	0.01 [-0.41, 0.42]
Romdhani 2019 (2)	-105.56	90.56	14	0	94.44	14	3.0%	-1.11 [-1.91, -0.30]
Skurvydas 2020-CMJ	0	0.07	15	0	0.05	15	3.3%	0.00 [-0.72, 0.72]
Souissi 2003am	0.1	0.82	13	0	0.7	13	3.1%	0.13 [-0.64, 0.90]
Souissi 2013 (1)am-PP	-0.21	1.5	12	0	1.46	12	3.1%	-0.14 [-0.94, 0.66]
Souissi 2013 (2)am-PP	-0.51	1.36	12	0	1.46	12	3.0%	-0.35 [-1.16, 0.46]
Taheri 2012	0.3	1.97	18	0	1.6	18	3.4%	0.16 [-0.49, 0.82]
**Subtotal (95% CI)**			**225**			**225**	**47.7%**	**-0.28 [-0.60, 0.04]**
Heterogeneity: Tau² = 0.25; Chi² = 39.44, df = 15 (P = 0.0005); I² = 62%								
Test for overall effect: Z = 1.73 (P = 0.08)								
**2.3.2 p.m.**								
Abedelmalek 2013pm	-2.4	0.79	12	0	0.3	12	1.7%	-3.88 [-5.32, -2.44]
Abedelmalek 2014	-1.71	0.85	36	0	0.97	36	3.7%	-1.85 [-2.41, -1.30]
Daaloul 2019-CMJ	-1.2	4.47	13	0	4.2	13	3.1%	-0.27 [-1.04, 0.50]
Daaloul 2019-SJ	-1.6	4.3	13	0	4.4	13	3.1%	-0.36 [-1.13, 0.42]
Rae 2017	-18	37.0405184	16	-5	42.142615	16	3.3%	-0.32 [-1.02, 0.38]
Romdhani 2019 (1)	-27.78	95.86	14	0	94.44	14	3.2%	-0.28 [-1.03, 0.46]
Romdhani 2020	-92.02	55.31	9	0	57.06	9	2.4%	-1.56 [-2.65, -0.47]
Saddoud 2023-SMBT	-0.36	0.65	24	0	0.66	24	3.6%	-0.54 [-1.12, 0.04]
Saddoud 2023-VJ	-3.96	6.26	24	0	6.47	24	3.6%	-0.61 [-1.19, -0.03]
Skein2011	0.02	0.15	10	0	0.14	7	2.6%	0.13 [-0.84, 1.10]
Souissi 2003pm	-0.4	0.66	13	0	0.7	13	3.1%	-0.57 [-1.36, 0.22]
Souissi 2013 (1)pm-PP	-0.21	1.42	12	0	1.33	12	3.1%	-0.15 [-0.95, 0.65]
Souissi 2013 (2)pm-PP	-1.08	1.26	12	0	1.33	12	3.0%	-0.80 [-1.64, 0.03]
Vardar 2007 (1)	-0.25	1.51	13	0	1.4	13	3.1%	-0.17 [-0.94, 0.60]
Vardar 2007 (2)	-0.4	1.4	13	0	1.4	13	3.1%	-0.28 [-1.05, 0.50]
**Subtotal (95% CI)**			**234**			**231**	**45.8%**	**-0.68 [-1.06, -0.31]**
Heterogeneity: Tau² = 0.38; Chi² = 49.89, df = 14 (P < 0.00001); I² = 72%								
Test for overall effect: Z = 3.57 (P = 0.0004)								
**2.3.3 without reference to**								
HajSalem 2013	-0.45	0.98	21	-0.475	0.93	21	3.6%	0.03 [-0.58, 0.63]
Mah 2019	-0.02	0.095	11	0	0.09	11	3.0%	-0.21 [-1.05, 0.63]
**Subtotal (95% CI)**			**32**			**32**	**6.5%**	**-0.05 [-0.54, 0.44]**
Heterogeneity: Tau² = 0.00; Chi² = 0.20, df = 1 (P = 0.66); I² = 0%								
Test for overall effect: Z = 0.22 (P = 0.83)								
**Total (95% CI)**			**491**			**488**	**100.0%**	**-0.46 [-0.70, -0.21]**
Heterogeneity: Tau² = 0.33; Chi² = 103.35, df = 32 (P < 0.00001); I² = 69%								
Test for overall effect: Z = 3.70 (P = 0.0002)								
Test for subgroup differences: Chi² = 4.53, df = 2 (P = 0.10), I² = 55.9%								

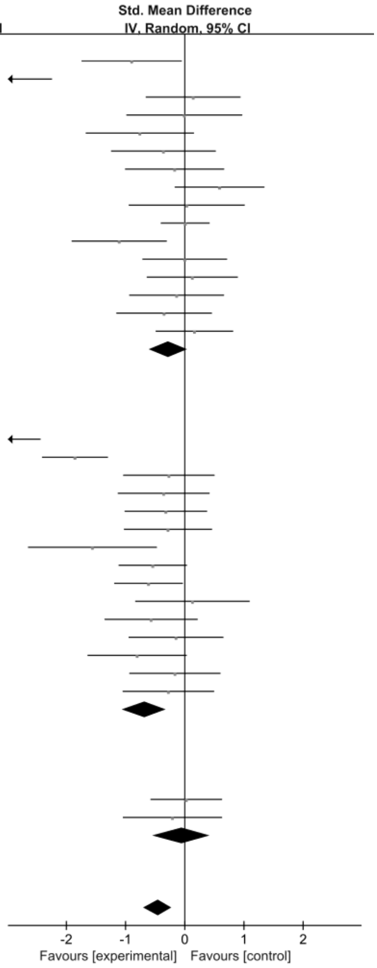

S6 Fig Forest plot of the effects of different test period of sleep deprivation on explosive power. am: ante meridiem; pm: post meridiem; CMJ: Counter Movement Jump; SJ, Squat Jump; VJ, Vertical Jumps; PP: Wingate peak power; SMBT: seated medicine-ball throw

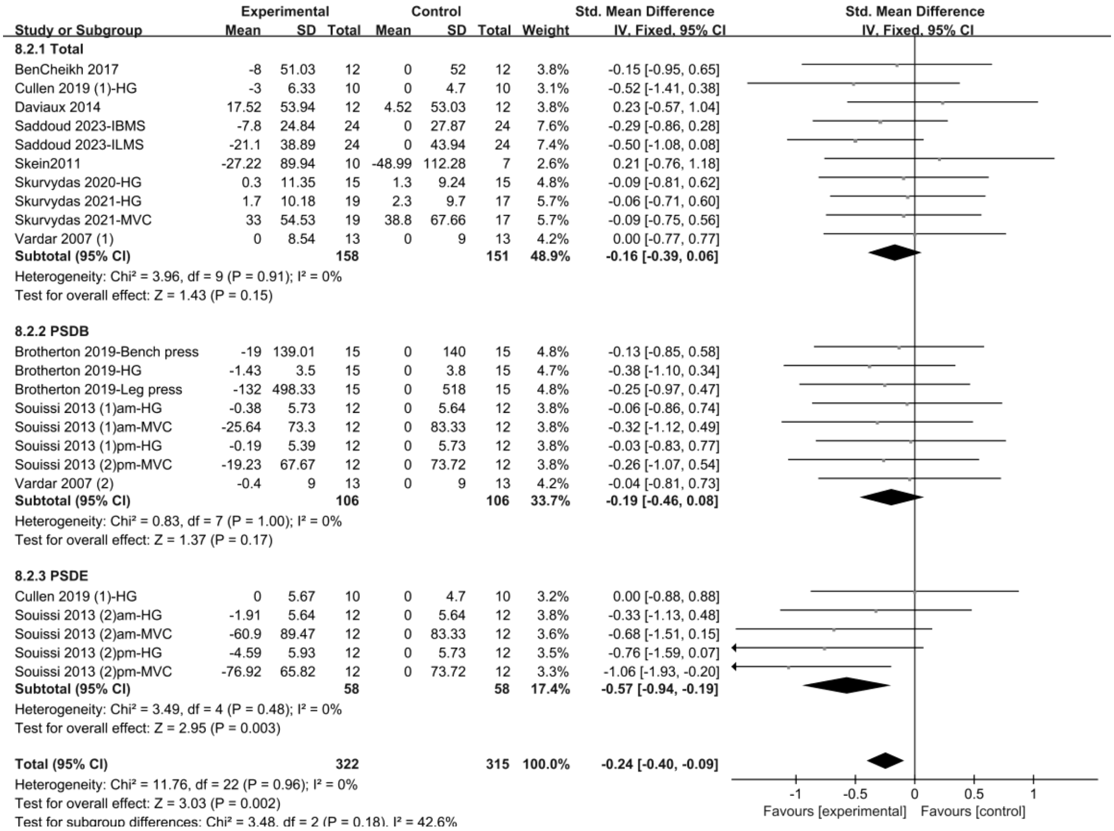

S7 Fig Forest plot of the effects of different types of sleep deprivation on maximum force. am: ante meridiem; pm: post meridiem; MVC: Maximal Isometric Voluntary Contraction; HG: Handgrip Strength; ILMS: isometric leg-muscles strength; IBMS: isometric back-muscles strength

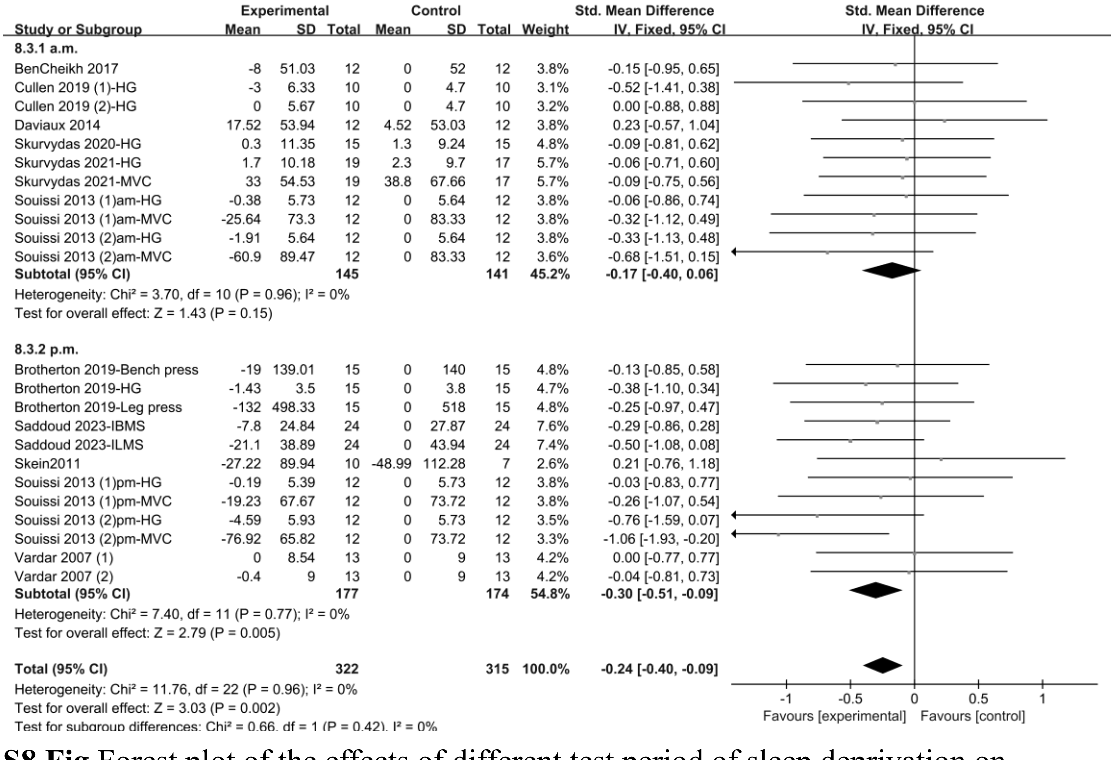

S8 Fig Forest plot of the effects of different test period of sleep deprivation on maximum force. am: ante meridiem; pm: post meridiem; MVC: Maximal Isometric

Voluntary Contraction; HG: Handgrip Strength; ILMS: isometric leg-muscles strength; IBMS: isometric back-muscles strength

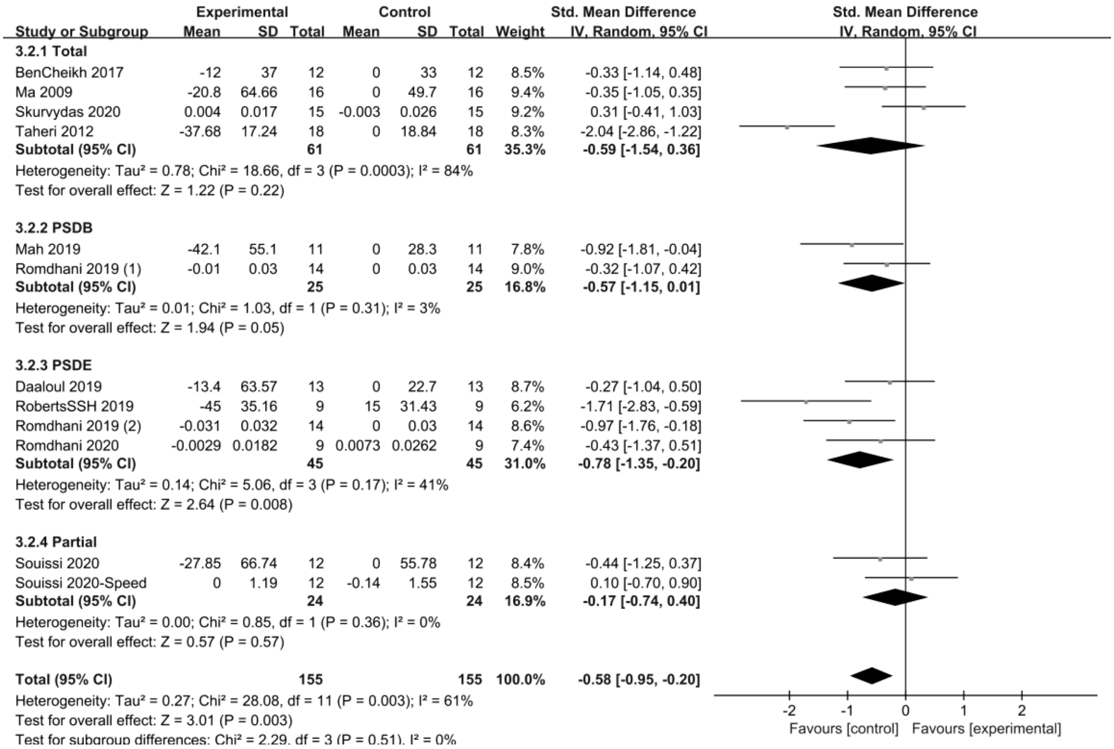

S9 Fig Forest plot of the effects of different types of sleep deprivation on speed performance

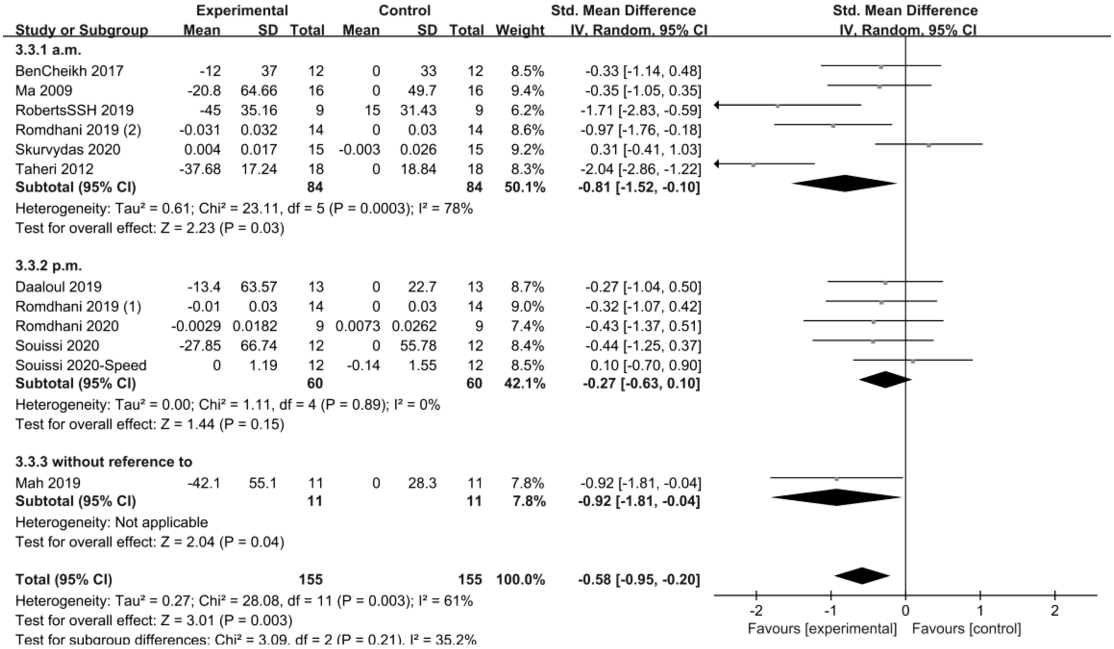

S10 Fig Forest plot of the effects of different test period of sleep deprivation on speed performance

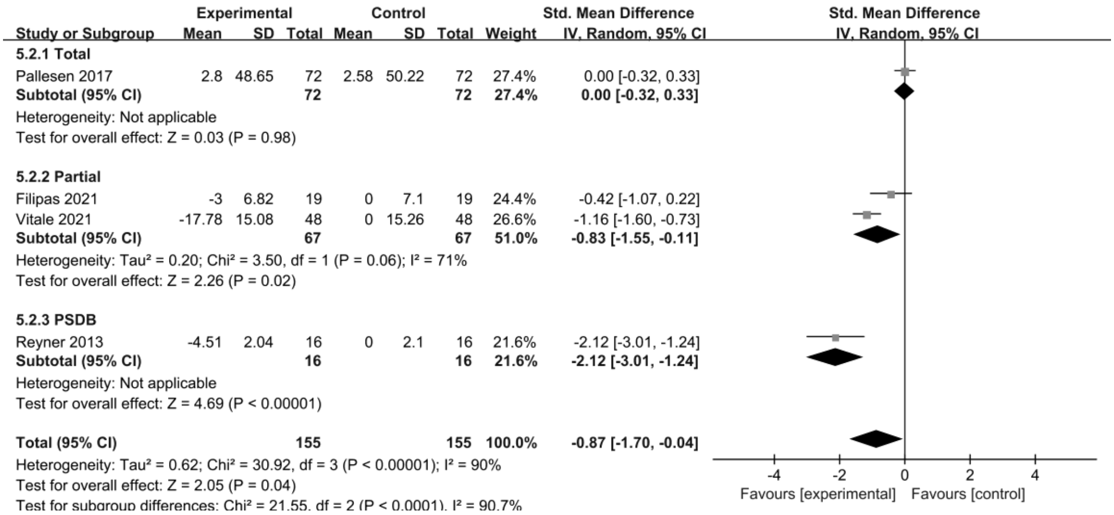

S11 Fig Forest plot of the effects of different types of sleep deprivation on skill control

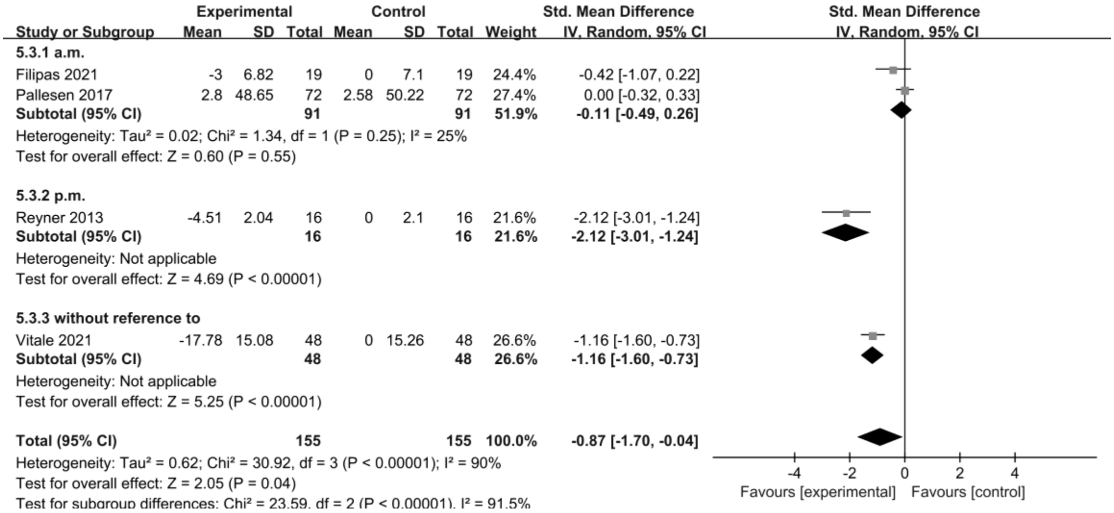

S12 Fig Forest plot of the effects of different test period of sleep deprivation on skill control

Study or Subgroup	Experimental Mean	Experimental SD	Experimental Total	Control Mean	Control SD	Control Total	Weight	Std. Mean Difference IV, Random, 95% CI
**6.2.1 Total**								
Blumert 2007	4	5.8	9	5.9	5.26	9	4.7%	-0.33 [-1.26, 0.60]
Daanen 2013	0.27	1.34	11	0	1.01	11	5.1%	0.22 [-0.62, 1.06]
Ma 2009	2.39	0.93	16	0	0.8	16	4.5%	2.69 [1.70, 3.67]
Skein2011	0.85	0.99	10	1.21	1.25	7	4.5%	-0.31 [-1.28, 0.66]
Skein 2013	-0.6	1.63707055	11	0	1.4	11	5.1%	-0.38 [-1.22, 0.47]
**Subtotal (95% CI)**			**57**			**54**	**23.9%**	**0.37 [-0.72, 1.45]**
Heterogeneity: Tau² = 1.31; Chi² = 28.22, df = 4 (P < 0.0001); I² = 86%								
Test for overall effect: Z = 0.66 (P = 0.51)								
**6.2.2 Partial**								
Knowles 2022	0.21	0.82	14	-0.6	0.97	14	5.4%	0.88 [0.09, 1.66]
Souissi 2020	1.4	3.89	12	0	3.97	12	5.2%	0.34 [-0.46, 1.15]
**Subtotal (95% CI)**			**26**			**26**	**10.6%**	**0.62 [0.06, 1.18]**
Heterogeneity: Tau² = 0.00; Chi² = 0.86, df = 1 (P = 0.35); I² = 0%								
Test for overall effect: Z = 2.16 (P = 0.03)								
**6.2.3 PSDB**								
Dean 2023	0.27	1.34	10	0	1.01	10	4.9%	0.22 [-0.66, 1.10]
Mejri 2016 (1)	0.36	2.85	10	0	3.15	10	4.9%	0.11 [-0.76, 0.99]
Romdhani 2019 (1)	0.84	0.67	14	0	0.55	14	5.1%	1.33 [0.50, 2.16]
Souissi 2013 (1)am-MP	0.18	1.61	12	0	1.06	12	5.3%	0.13 [-0.67, 0.93]
Souissi 2013 (1)pm-MP	0.79	1.83	12	0	0.97	12	5.2%	0.52 [-0.30, 1.34]
**Subtotal (95% CI)**			**58**			**58**	**25.5%**	**0.47 [0.02, 0.92]**
Heterogeneity: Tau² = 0.08; Chi² = 5.78, df = 4 (P = 0.22); I² = 31%								
Test for overall effect: Z = 2.03 (P = 0.04)								
**6.2.4 PSDE**								
Ajjimaporn 2020	1	1	11	0	1	11	4.9%	0.96 [0.07, 1.85]
Chase 2017	0.3	1.04	8	0	0.89	8	4.5%	0.29 [-0.69, 1.28]
HajSalem 2013	1	15.42	21	0	14.9	21	6.2%	0.06 [-0.54, 0.67]
Mejri 2016 (2)	0.11	3.02	10	0	3.15	10	4.9%	0.03 [-0.84, 0.91]
Romdhani 2019 (2)	0.45	0.58	14	0	0.55	14	5.4%	0.77 [0.00, 1.55]
Romdhani 2020	0.24	0.66	9	0	0.67	9	4.7%	0.34 [-0.59, 1.28]
Souissi 2013 (2)am-MP	0.79	1.43	12	0	1.06	12	5.2%	0.61 [-0.22, 1.43]
Souissi 2013(2)pm-MP	2.64	1.42	12	0	0.97	12	4.3%	2.10 [1.07, 3.13]
**Subtotal (95% CI)**			**97**			**97**	**40.1%**	**0.60 [0.17, 1.02]**
Heterogeneity: Tau² = 0.19; Chi² = 14.06, df = 7 (P = 0.05); I² = 50%								
Test for overall effect: Z = 2.77 (P = 0.006)								
**Total (95% CI)**			**238**			**235**	**100.0%**	**0.51 [0.20, 0.82]**
Heterogeneity: Tau² = 0.31; Chi² = 50.07, df = 19 (P = 0.0001); I² = 62%								
Test for overall effect: Z = 3.23 (P = 0.001)								
Test for subgroup differences: Chi² = 0.34, df = 3 (P = 0.95), I² = 0%								

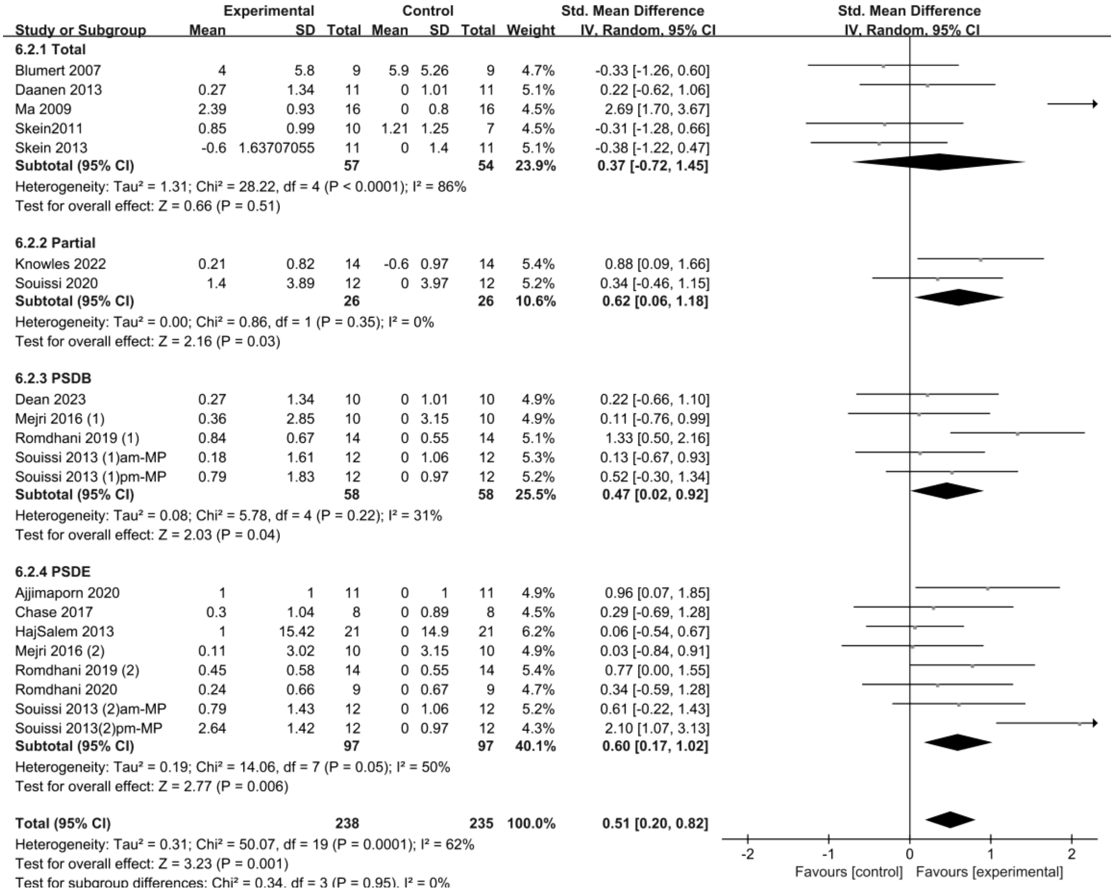

S13 Fig Forest plot of the effects of different types of sleep deprivation on rating of perceived exertion. am: ante meridiem; pm: post meridiem; MP: Wingate mean power

Study or Subgroup	Experimental Mean	Experimental SD	Experimental Total	Control Mean	Control SD	Control Total	Weight	Std. Mean Difference IV, Random, 95% CI
**6.3.1 a.m.**								
Ajjimaporn 2020	1	1	11	0	1	11	4.9%	0.96 [0.07, 1.85]
Blumert 2007	4	5.8	9	5.9	5.26	9	4.7%	-0.33 [-1.26, 0.60]
Chase 2017	0.3	1.04	8	0	0.89	8	4.5%	0.29 [-0.69, 1.28]
Daanen 2013	0.27	1.34	11	0	1.01	11	5.1%	0.22 [-0.62, 1.06]
Dean 2023	0.27	1.34	10	0	1.01	10	4.9%	0.22 [-0.66, 1.10]
Knowles 2022	0.21	0.82	14	-0.6	0.97	14	5.4%	0.88 [0.09, 1.66]
Ma 2009	2.39	0.93	16	0	0.8	16	4.5%	2.69 [1.70, 3.67]
Mejri 2016 (1)	0.36	2.85	10	0	3.15	10	4.9%	0.11 [-0.76, 0.99]
Mejri 2016 (2)	0.11	3.02	10	0	3.15	10	4.9%	0.03 [-0.84, 0.91]
Skein 2013	-0.6	1.63707055	11	0	1.4	11	5.1%	-0.38 [-1.22, 0.47]
Souissi 2013 (1)am-MP	0.18	1.61	12	0	1.06	12	5.3%	0.13 [-0.67, 0.93]
Souissi 2013 (2)am-MP	0.79	1.43	12	0	1.06	12	5.2%	0.61 [-0.22, 1.43]
**Subtotal (95% CI)**			**134**			**134**	**59.3%**	**0.44 [0.01, 0.86]**
Heterogeneity: Tau² = 0.36; Chi² = 31.28, df = 11 (P = 0.0010); I² = 65%								
Test for overall effect: Z = 2.03 (P = 0.04)								
**6.3.2 p.m.**								
Romdhani 2019 (1)	0.84	0.67	14	0	0.55	14	5.1%	1.33 [0.50, 2.16]
Romdhani 2019 (2)	0.45	0.58	14	0	0.55	14	5.4%	0.77 [0.00, 1.55]
Romdhani 2020	0.24	0.66	9	0	0.67	9	4.7%	0.34 [-0.59, 1.28]
Skein2011	0.85	0.99	10	1.21	1.25	7	4.5%	-0.31 [-1.28, 0.66]
Souissi 2013 (1)pm-MP	0.79	1.83	12	0	0.97	12	5.2%	0.52 [-0.30, 1.34]
Souissi 2013(2)pm-MP	2.64	1.42	12	0	0.97	12	4.3%	2.10 [1.07, 3.13]
Souissi 2020	1.4	3.89	12	0	3.97	12	5.2%	0.34 [-0.46, 1.15]
**Subtotal (95% CI)**			**83**			**80**	**34.5%**	**0.72 [0.20, 1.24]**
Heterogeneity: Tau² = 0.29; Chi² = 14.94, df = 6 (P = 0.02); I² = 60%								
Test for overall effect: Z = 2.70 (P = 0.007)								
**6.3.3 without reference to**								
HajSalem 2013	1	15.42	21	0	14.9	21	6.2%	0.06 [-0.54, 0.67]
**Subtotal (95% CI)**			**21**			**21**	**6.2%**	**0.06 [-0.54, 0.67]**
Heterogeneity: Not applicable								
Test for overall effect: Z = 0.21 (P = 0.83)								
**Total (95% CI)**			**238**			**235**	**100.0%**	**0.51 [0.20, 0.82]**
Heterogeneity: Tau² = 0.31; Chi² = 50.07, df = 19 (P = 0.0001); I² = 62%								
Test for overall effect: Z = 3.23 (P = 0.001)								
Test for subgroup differences: Chi² = 2.56, df = 2 (P = 0.28), I² = 22.0%								

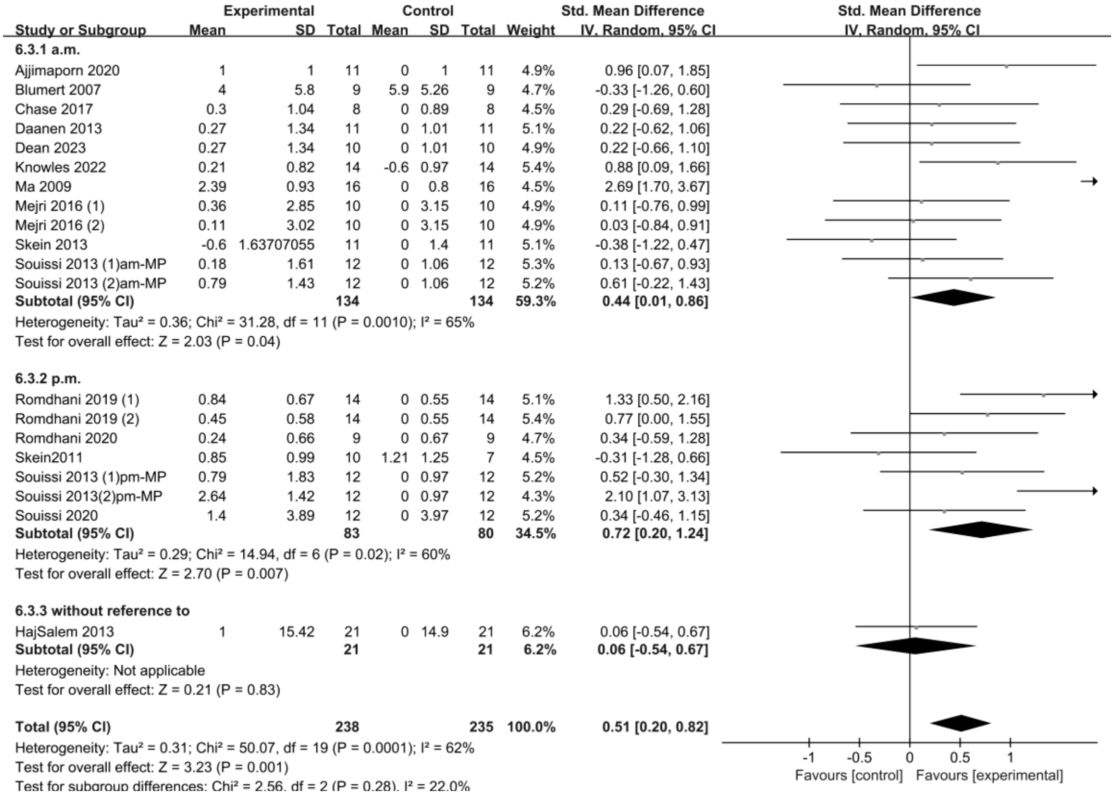

S14 Fig Forest plot of the effects of different test period of sleep deprivation on rating of perceived exertion. am: ante meridiem; pm: post meridiem; MP: Wingate mean power

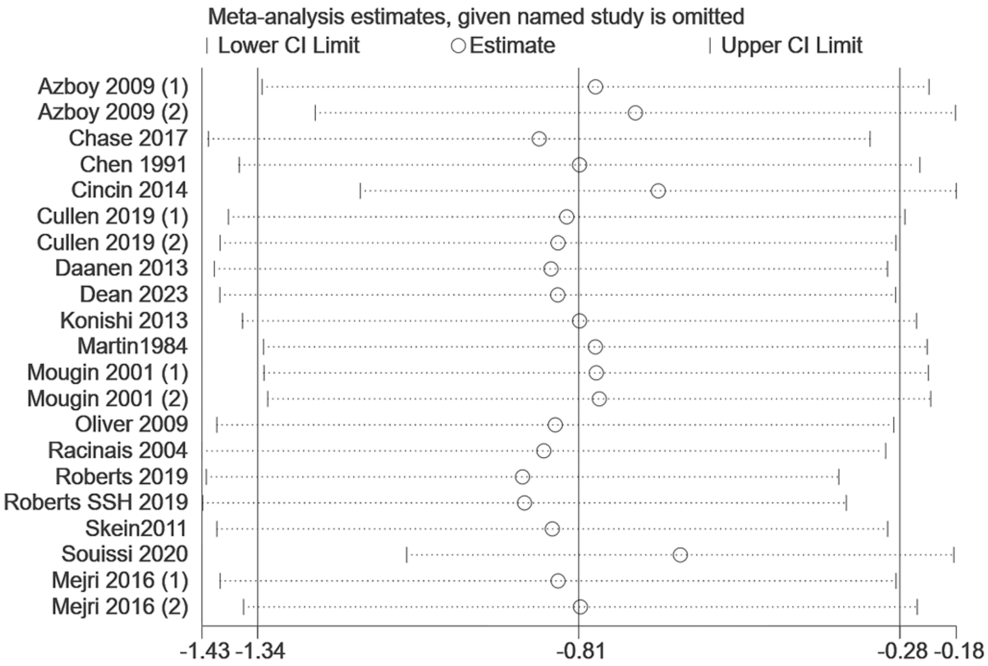

S15 Fig Sensitivity analysis plot of aerobic endurance performance

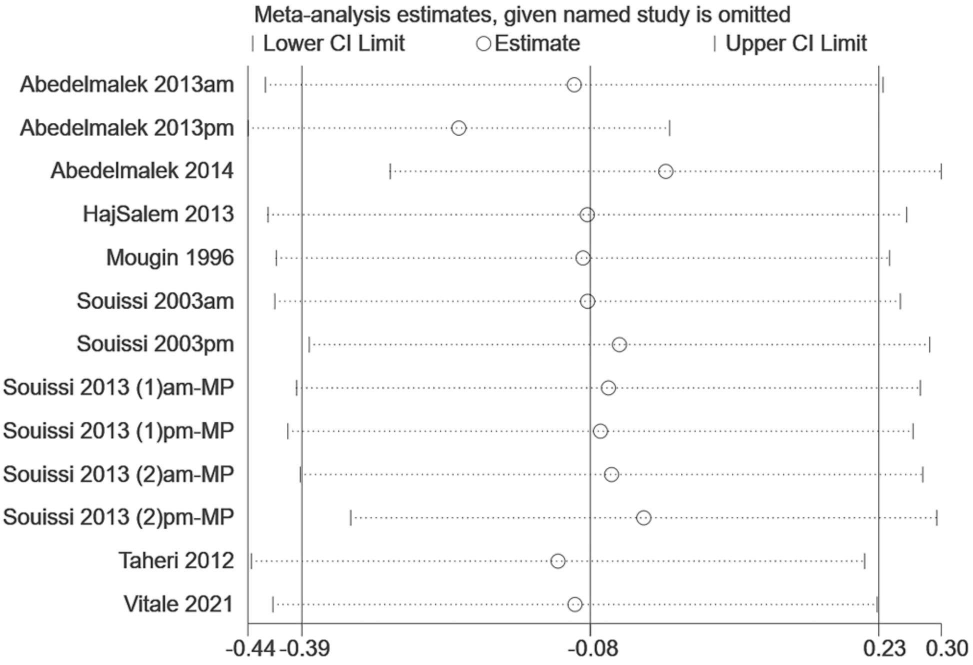

S16 Fig Sensitivity analysis plot of anerobic endurance performance. am: ante meridiem; pm: post meridiem; MP: Wingate mean power

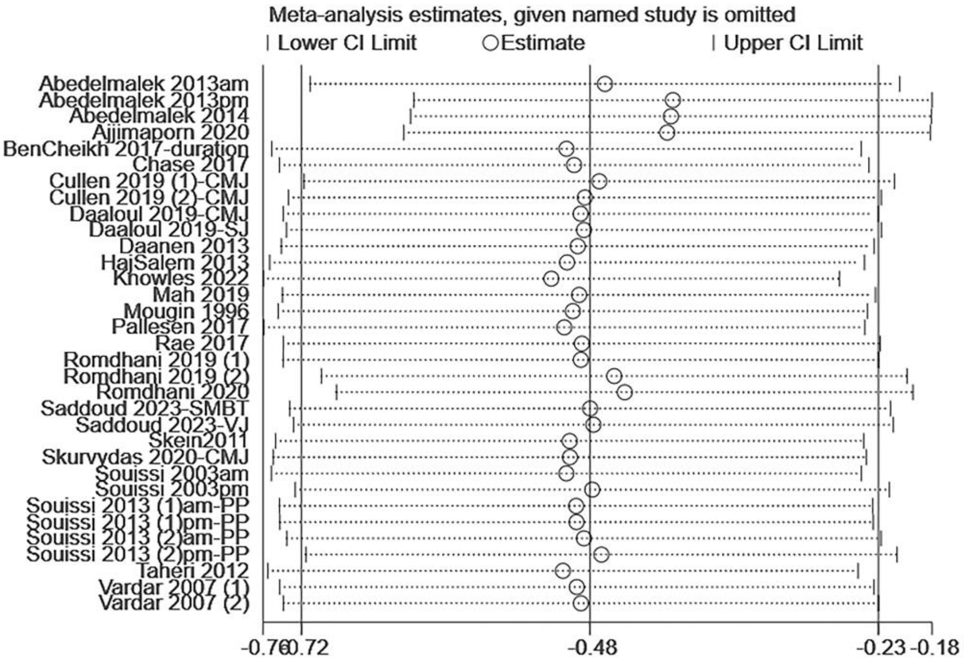

S17 Fig Sensitivity analysis plot of explosive power. am: ante meridiem; pm: post meridiem; CMJ: Counter Movement Jump; SJ, Squat Jump; VJ, Vertical Jumps; PP: Wingate peak power; SMBT: seated medicine-ball throw

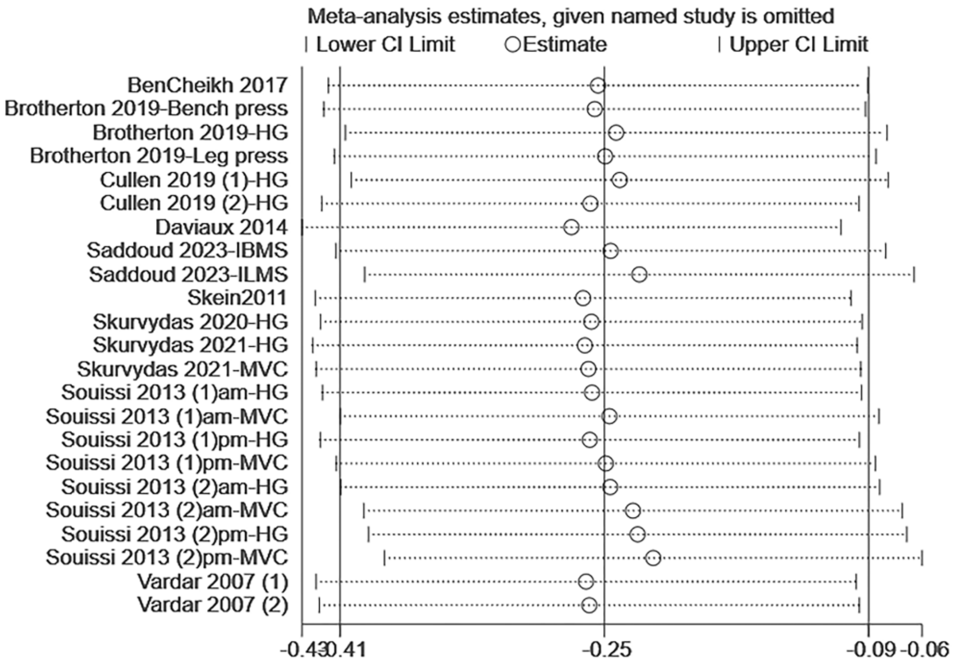

S18 Fig Sensitivity analysis plot of maximum force. am: ante meridiem; pm: post meridiem; MVC: Maximal Isometric Voluntary Contraction; HG: Handgrip Strength; ILMS: isometric leg-muscles strength; IBMS: isometric back-muscles strength

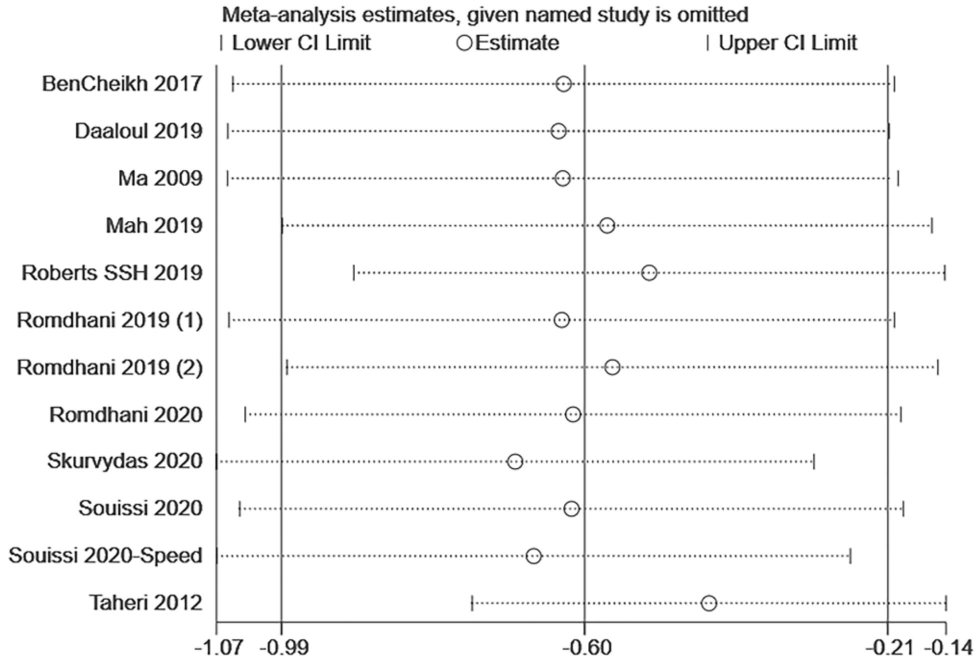

S19 Fig Sensitivity analysis plot of speed performance

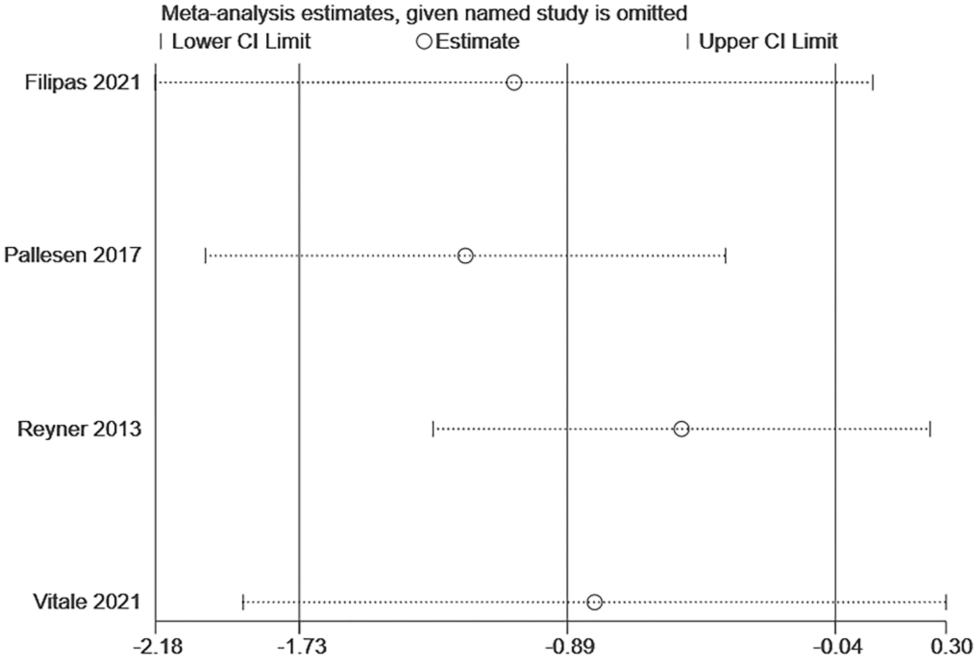

S20 Fig Sensitivity analysis plot of skill control

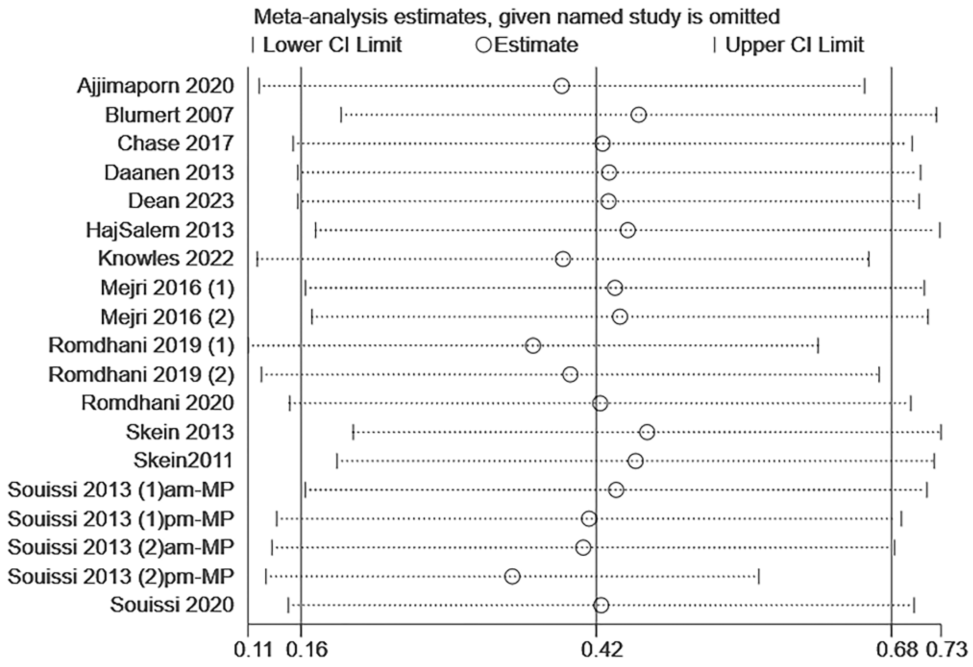

S21 Fig Sensitivity analysis plots of rating of perceived exertion. am: ante meridiem; pm: post meridiem; MP: Wingate mean power

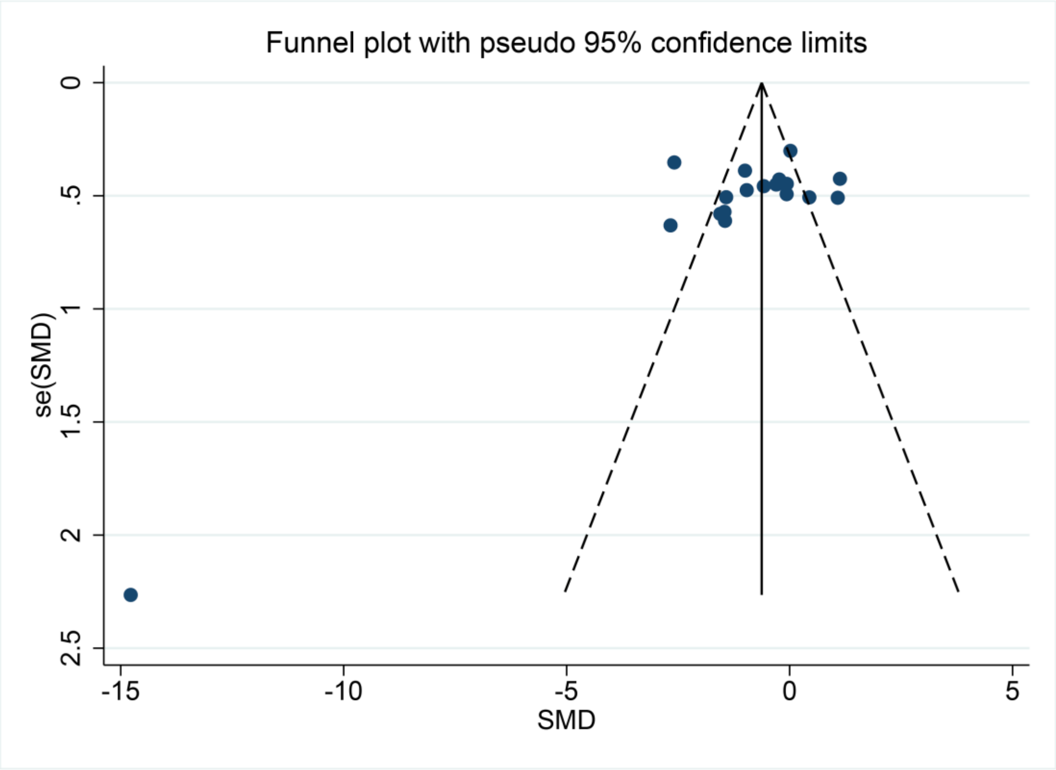

S22 Fig Funnel plot of aerobic endurance performance

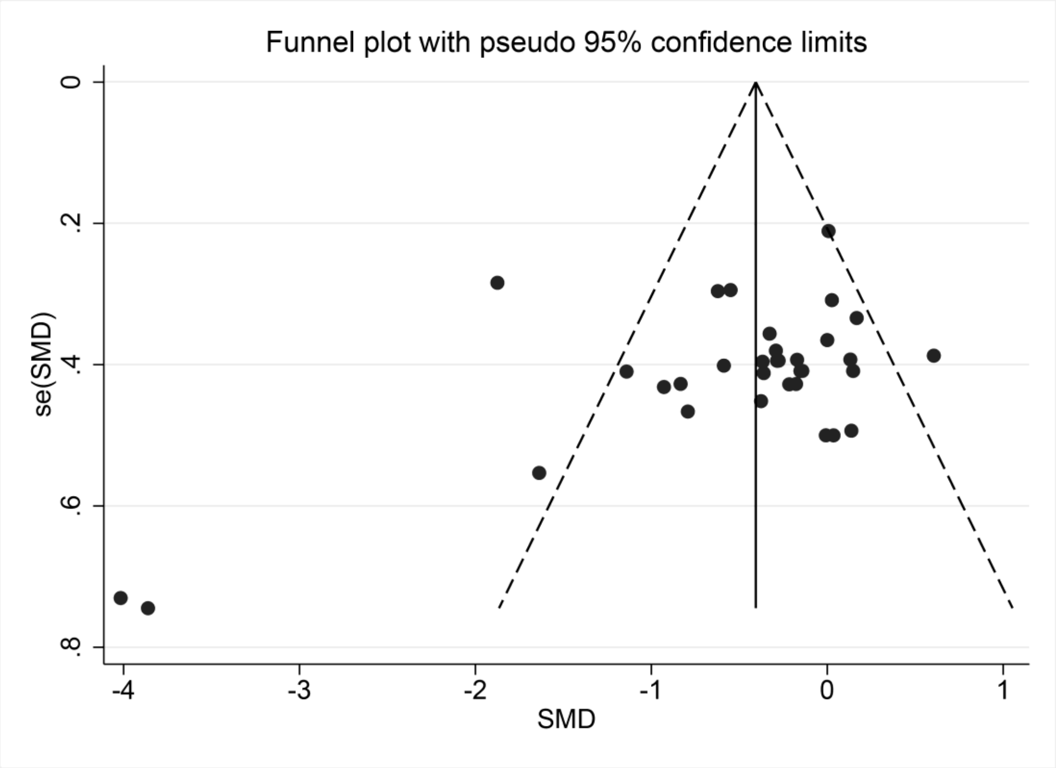

S23 Fig Funnel plot of explosive power

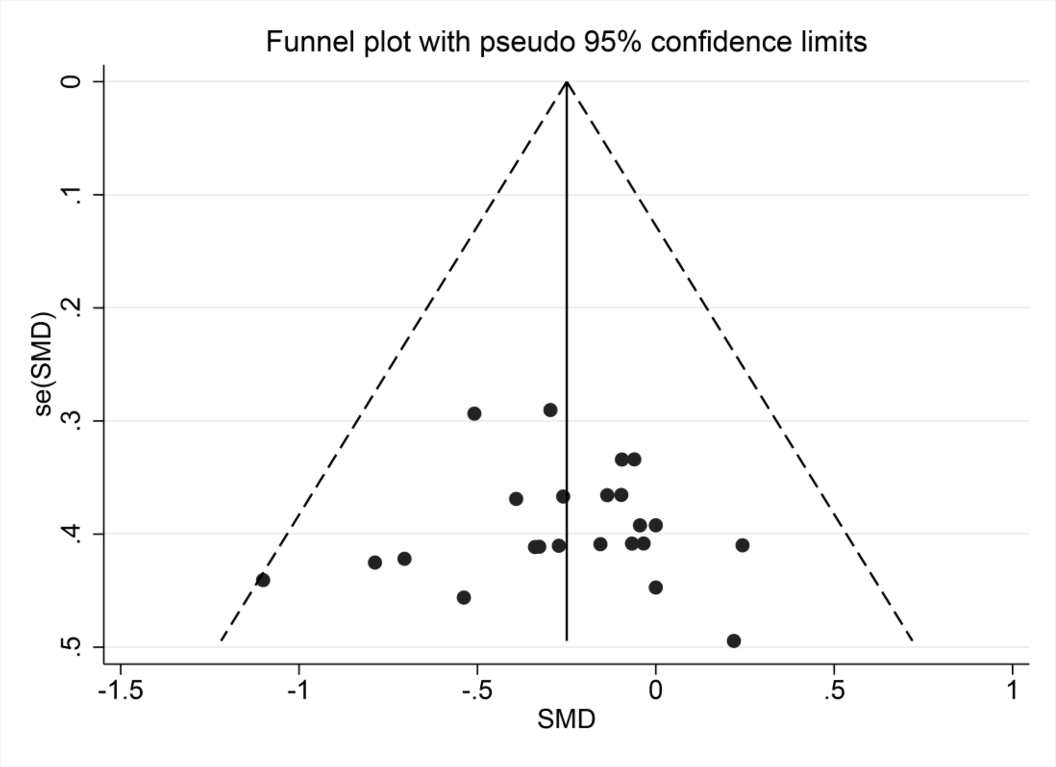

S24 Fig Funnel plot of maximum force

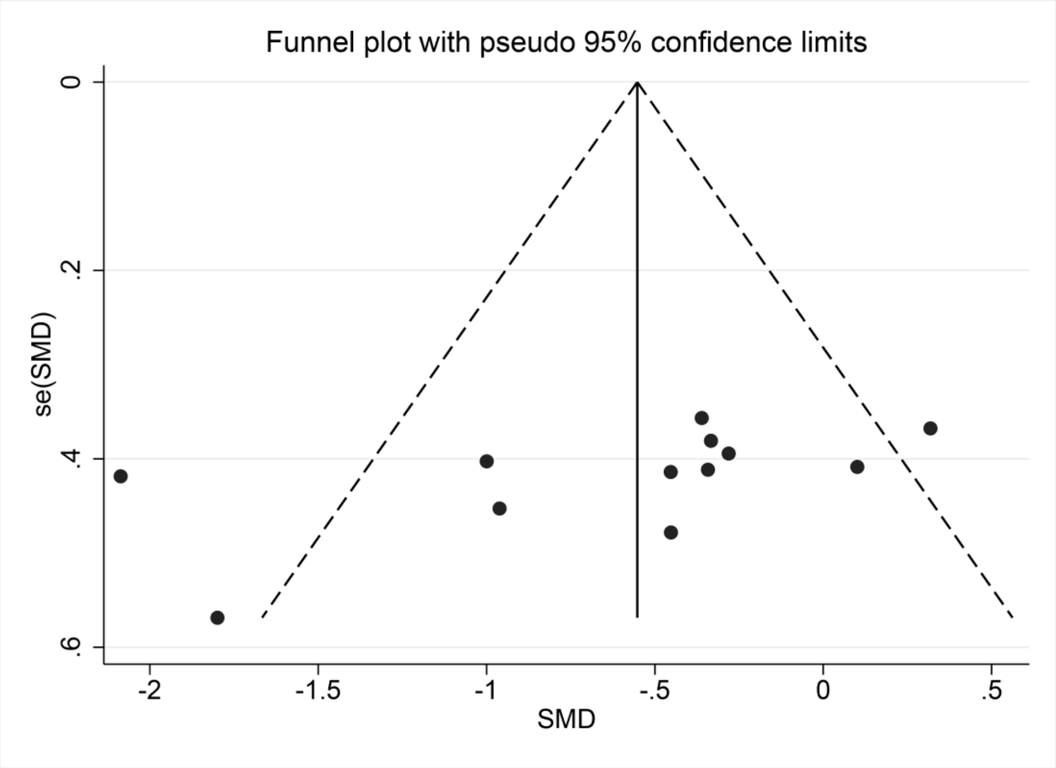

S25 Fig Funnel plot of speed performance

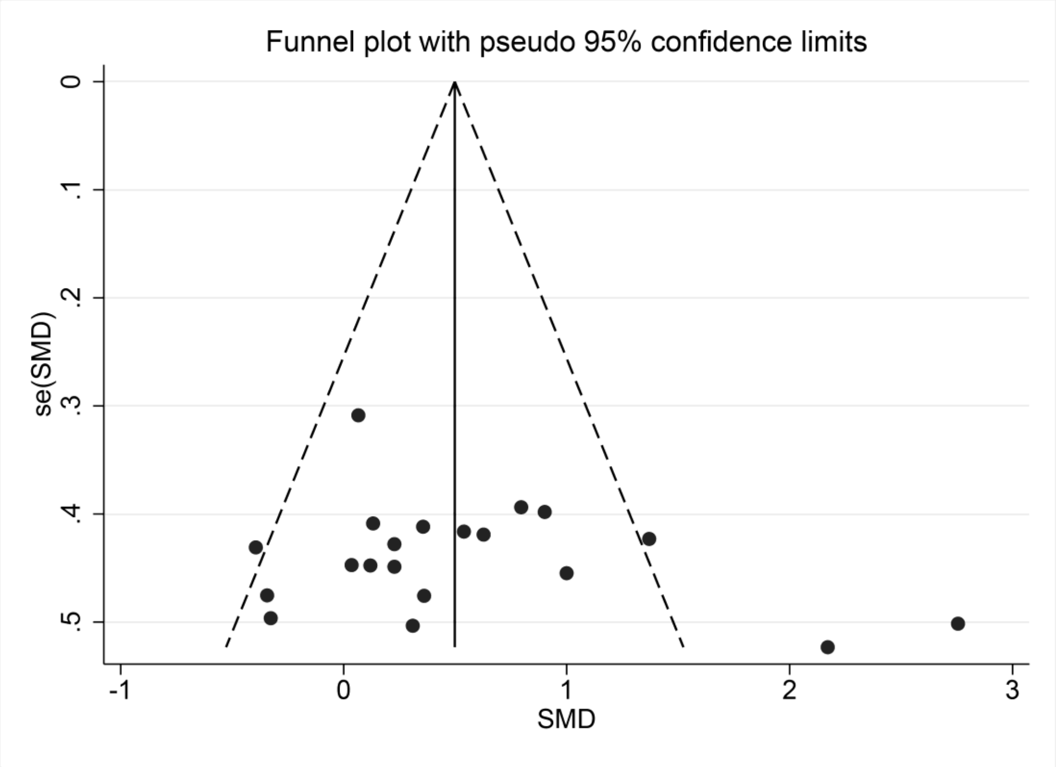

S26 Fig Funnel plot of rating of perceived exertion

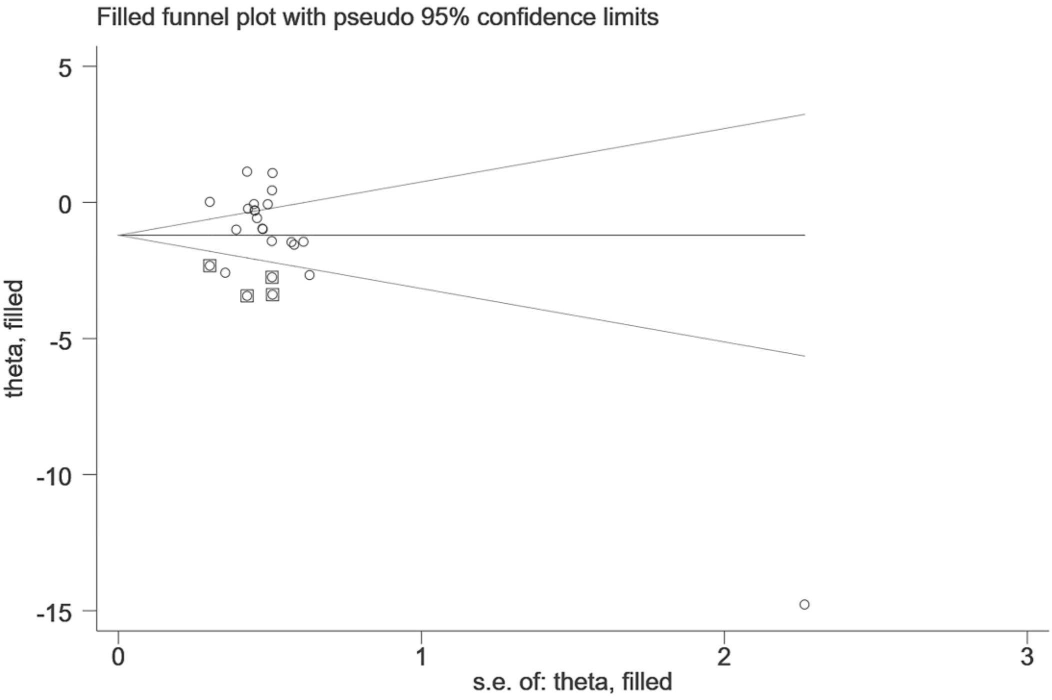

S27 Fig Funnel plot of aerobic endurance performance after trimming and filling
